# Longer-term and landmark analysis of transcatheter vs. surgical aortic-valve implantation in severe aortic stenosis: a meta-analysis

**DOI:** 10.3389/fcvm.2025.1479200

**Published:** 2025-03-06

**Authors:** Yu Wang, Xiaowen Zhang, Xinlin Zhang, Wei Xu

**Affiliations:** ^1^ Department of Cardiology, Nanjing Drum Tower Hospital, The Affiliated Hospital of Nanjing University Medical School, Nanjing, China; ^2^ Endocrine and Metabolic Disease Medical Center, Nanjing Drum Tower Hospital, The Affiliated Hospital of Nanjing University Medical School, Nanjing, China

**Keywords:** aortic stenosis, TAVI, SAVR, longer-term, randomized controlled trials (RCT)

## Abstract

**Background:**

Previous reports of longer-term outcomes of transcatheter aortic valve implantation (TAVI) focus on higher risk patients and suggest potential temporal changes.

**Aims:**

To evaluate the longer-term and temporal performances of TAVI compared to surgical aortic valve replacement (SAVR).

**Methods:**

Randomized controlled trials reporting outcomes with at least 1-year follow-up. The primary outcome was the composite of all-cause death or disabling stroke.

**Results:**

We included 8 trials with 8,749 patients. TAVI was associated with a higher risk of longer-term (5-year) primary outcome compared to SAVR among higher-risk [odds ratio (OR), 1.25; 95% CI, 1.07–1.47] but not lower-risk participants [1.0 (0.77–1.29)]. However, a significant temporal interaction was detected in both risk profiles. TAVI with balloon-expandable valves was associated with a higher risk of longer-term primary outcome compared to SAVR [1.38 (1.2–1.6)], whereas no statistical difference was found with self-expanding valves [1.03 (0.89–1.19)]. There was a significant interaction between the two valve systems, and a temporal interaction was detected in both systems. Overall landmark analysis revealed a lower risk in TAVI within the initial 30 days [0.76 (0.6, 0.96)], comparable between 30 days to 2 years [1.04 (0.85, 1.28)], and higher beyond 2 years [1.36 (1.15–1.61)]. Analysis for all-cause death generated largely similar results.

**Conclusions:**

TAVI was associated with a higher longer-term risk of primary outcome compared to SAVR in higher-risk patients and with balloon-expandable valves. However, a characteristic temporal interaction was documented in all subgroups. Future studies are warranted to test these findings.

## 1 Introduction

Transcatheter aortic valve implantation (TAVI) has emerged as a popular treatment for patients with severe aortic stenosis, surpassing surgical procedures in some countries (1). We previously indicated a potential higher mortality associated with TAVI compared to surgical aortic valve replacement (SAVR) at 5-year follow-up (2), mainly in high risk patients (3–5). The longer-term performance of TAVI vs. SAVR in patients with lower risk remains uncertain. Additionally, the temporal changes in TAVI performance at different timepoints have yet to be determined. Given the expansion of TAVI to low-risk patients with increased life expectancy, this assessment holds critical clinical importance.

The 5-year follow-up data from nearly all registered comparative randomized controlled trials (RCTs) of TAVI vs. SAVR have recently been published (6–9). We therefore are able to assess the longer-term outcomes of TAVI and conduct a landmark analysis to identify the timepoint at which the performance of TAVI might diverge from SAVR, as indicated in some studies (5). The aim of our study was to evaluate the longer-term and temporal performances of TAVI compared to SAVR, both overall and within important subgroups.

## 2 Methods

We reported the meta-analysis in accordance with the Preferred Reporting Items for Systematic Reviews and Meta-Analyses (PRISMA) guideline (Supplementary Table S1).

### 2.1 Data sources and searches

PubMed, the Cochrane Central Register of Controlled Trials, EMBASE, and major conference proceedings were systematically searched from inception through October 25, 2023, an update of our previous meta-analysis (2). The computer-based searches combined terms and keywords which included transcatheter aortic valve implantation, transcatheter aortic valve replacement, TAVI, TAVR, and randomized trial (Supplementary Materials). Two investigators independently hand-searched the references of identified studies and relevant reviews to identify any additional relevant trials.

### 2.2 Study selection

Two reviewers conducted independent screening of titles and abstracts to determine eligibility of the studies. Full-text articles were retrieved for studies that were deemed potentially relevant. In cases where discrepancies arose, a third investigator resolved the discrepancies. Eligible studies had to be RCTs evaluating TAVI vs. SAVR in patients with severe aortic stenosis, and reporting outcomes of interest with at least 1-year follow-up. Nonrandomized observational studies, studies comparing different types of TAVI devices, and studies with less than 1-year follow-up were excluded.

### 2.3 Outcome measures

The primary outcome was the composite of all-cause death and disabling stroke. Secondary outcomes included all-cause death, cardiovascular death, myocardial infarction, stroke, transient ischemic attack (TIA), major bleeding, major vascular complications (MVC), permanent pacemaker implantation (PPM), new-onset atrial fibrillation, aortic-valve reintervention, rehospitalization, and moderate or severe paravalvular leak (PVL).

### 2.4 Data extraction and quality assessment

Two investigators independently extracted the data using a pre-specified form. Whenever possible, data from the intention-to-treat analysis were extracted; otherwise, data from the as-treated analysis were extracted. The same investigators also assessed the risk of bias in the included RCTs using the Cochrane Risk of Bias 2.0 tool.

### 2.5 Statistical analysis

Summary measures were reported as odds ratios (ORs) and pooled using random-effects models (DerSimonian–Laird method). Data were analyzed separately for different time points, including data within 30 days, 1 year, 2 years, and 5 years (one trial reported 4-year outcome and was used), and categorized as early, short-term, midterm, and longer-term outcomes, respectively. Landmark analysis was also conducted for intervals within 1 year, between 1 year and 2 years, and beyond 2 years. Events occurring within 1 year were further divided into events within 30 days and events between 30 days and 1 year to further explore the timing of performance change. For trials in which only one of the arms had no events, the 0.5 continuity correction was applied. Stratified analyses were performed based on surgical risks (higher and lower risks) and TAVI systems [balloon-expandable valves (BEV) and self-expanding valves (SEV)]. The higher-risk group included trials involving patients with extreme, high, and intermediate-to-high surgical risk, while the lower-risk group included trials involving patients with low and low-to-intermediate risk, as determined by the evaluation using the Society of Thoracic Surgeons predicted risk of mortality (STS-PROM) score. Between-subgroup differences were assessed using the *χ*^2^-test for heterogeneity. Sensitivity analysis was performed for the primary outcomes using Hartung-Knapp-Sidik-Jonkman variance correction, and by removing an individual trial each time. Heterogeneity was evaluated using the Q and I2 statistics. All meta-analyses were performed using Stata software version 16.0, and the Review Manager version 5.3. A 2-tailed *p* value <0.05 was considered statistically significant.

## 3 Results

### 3.1 Study selection and characteristics

We included 8 trials and 14 secondary reports that provided eligible data from these trials (3–23), involving a total of 8,749 patients (Supplementary Figure S1). All 8 trials reported outcomes at 30 days and 1 year, 7 reported 2-year outcomes, one reported 4-year outcomes (8), and 6 reported 5-year outcomes (3–6, 9, 20). The mean age was 79.2 years and 57.4% were male. Based on STS-PROM risk score, 4 trials were categorized as lower-risk trials (mean STS PROM 1.9%–3.0%), while the other 4 categorized as higher-risk trials (mean STS PROM 4.5%–11.7%). BEV was used in 3 trials, SEV in 4 trials, and a mixed TAVI system in one trial. Baseline characteristics are presented in Supplementary Tables S2, S3. Blinding of participants and personnel was not feasible in any of the trials (Supplementary Table S4).

### 3.2 Primary outcome

**Figure 1:**
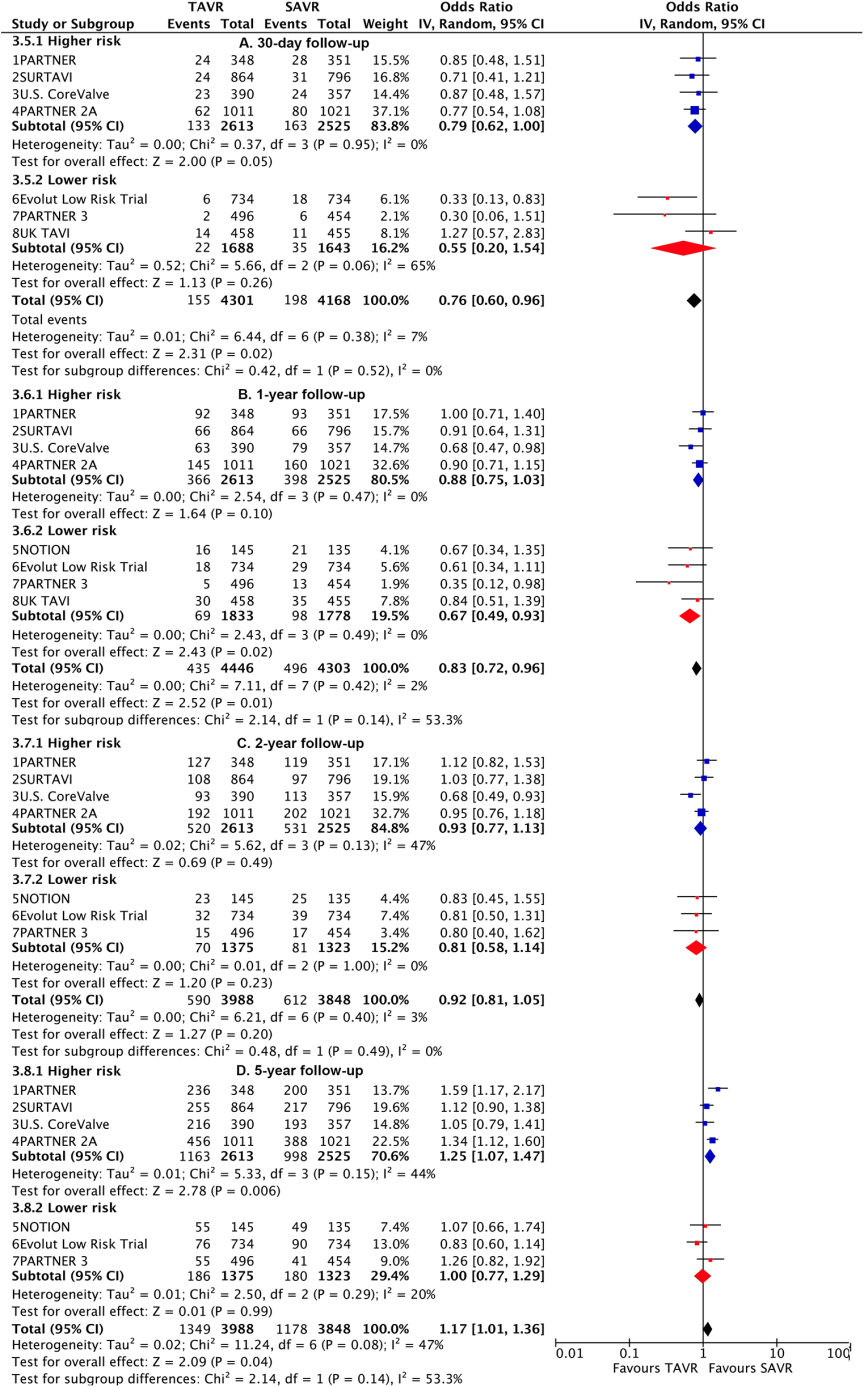
Risk estimates of all-cause death or disabling stroke for TAVI vs SAVR stratified by surgical risks at different lengths of follow-up.

**Figure 2:**
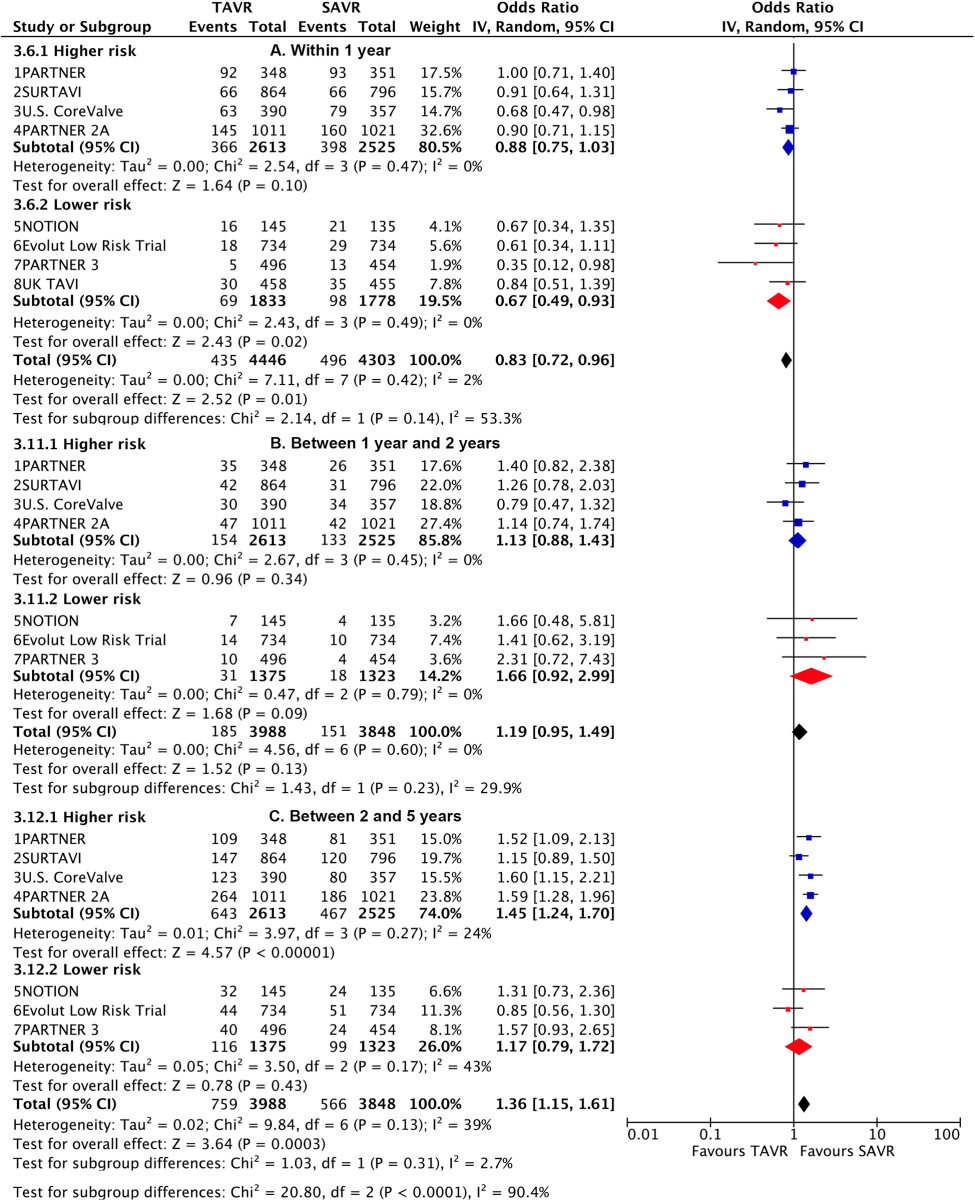
Landmark risk estimates of all-cause death or disabling stroke for TAVI vs SAVR stratified by surgical risks.

TAVI demonstrated a lower rate of primary outcome compared to SAVR at 30 days [odds ratio (OR), 0.76 (95% CI 0.6–0.96)] and 1 year [0.83 (0.72–0.96)]. However, at longer-term follow-up, TAVI was associated with a higher risk [1.17 (1.01–1.36)] (Figure 1). Landmark analysis indicated a significant benefit of TAVI within the first year, comparable events between 1 year and 2 years [1.19 (0.95–1.49)], but a significant disadvantage beyond 2 years [1.36 (1.15–1.61)], with a significant temporal interaction (p for interaction<0.0001) (Figure 2). The most notable benefit of TAVI was observed within the initial 30 days, whereas no significant difference was found between 30 days and 1 year [0.9 (0.74–1.08)] (Supplementary Table S5).

Subgroup analysis revealed a higher risk of longer-term primary outcome in TAVI compared to SAVR among participants with higher risk [1.25 (1.07–1.47)], but no statistical difference was found in patients with lower risk [1.00 (0.77–1.29)]. The higher risk of TAVI in higher-risk patients was primarily attributed to events occurring beyond 2 years [1.45 (1.24–1.7)] (p for interaction<0.0001) (Figure 2; Supplementary Table S6). The lower risk of TAVI over SAVR in lower-risk patients within 1 year [0.67 (0.49–0.93)] was not observed at longer-term follow-up, and a significant temporal interaction was detected (p for interaction = 0.01) (Supplementary Table S7).

**Figure 3:**
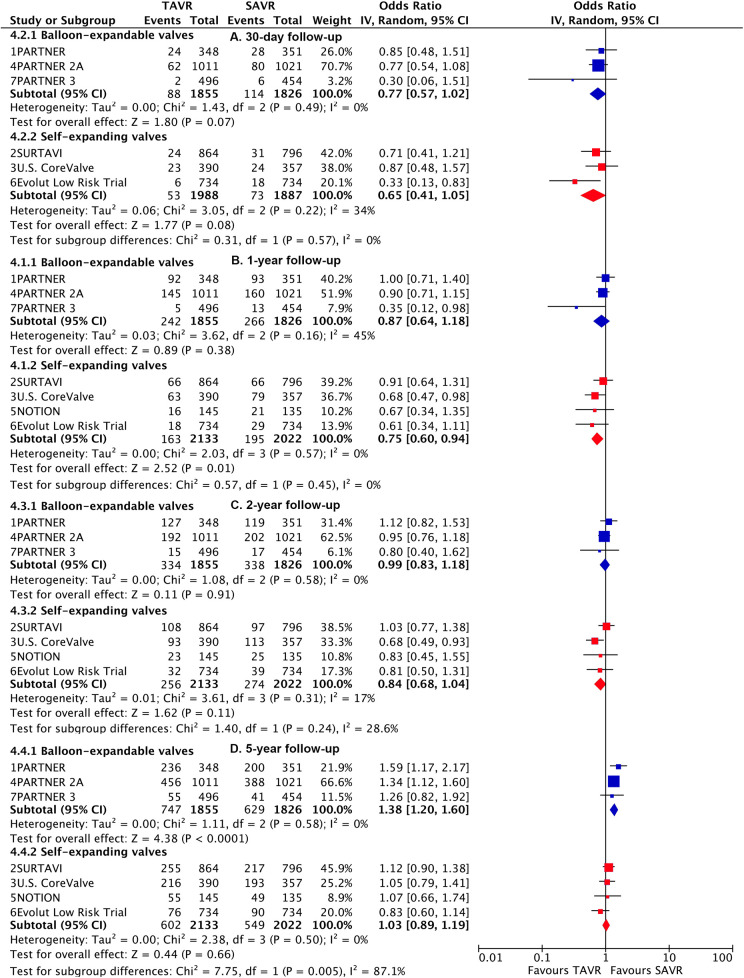
Risk estimates of all-cause death or disabling stroke for TAVI vs SAVR stratified by TAVI valve systems at different lengths of follow-up.

**Figure 4:**
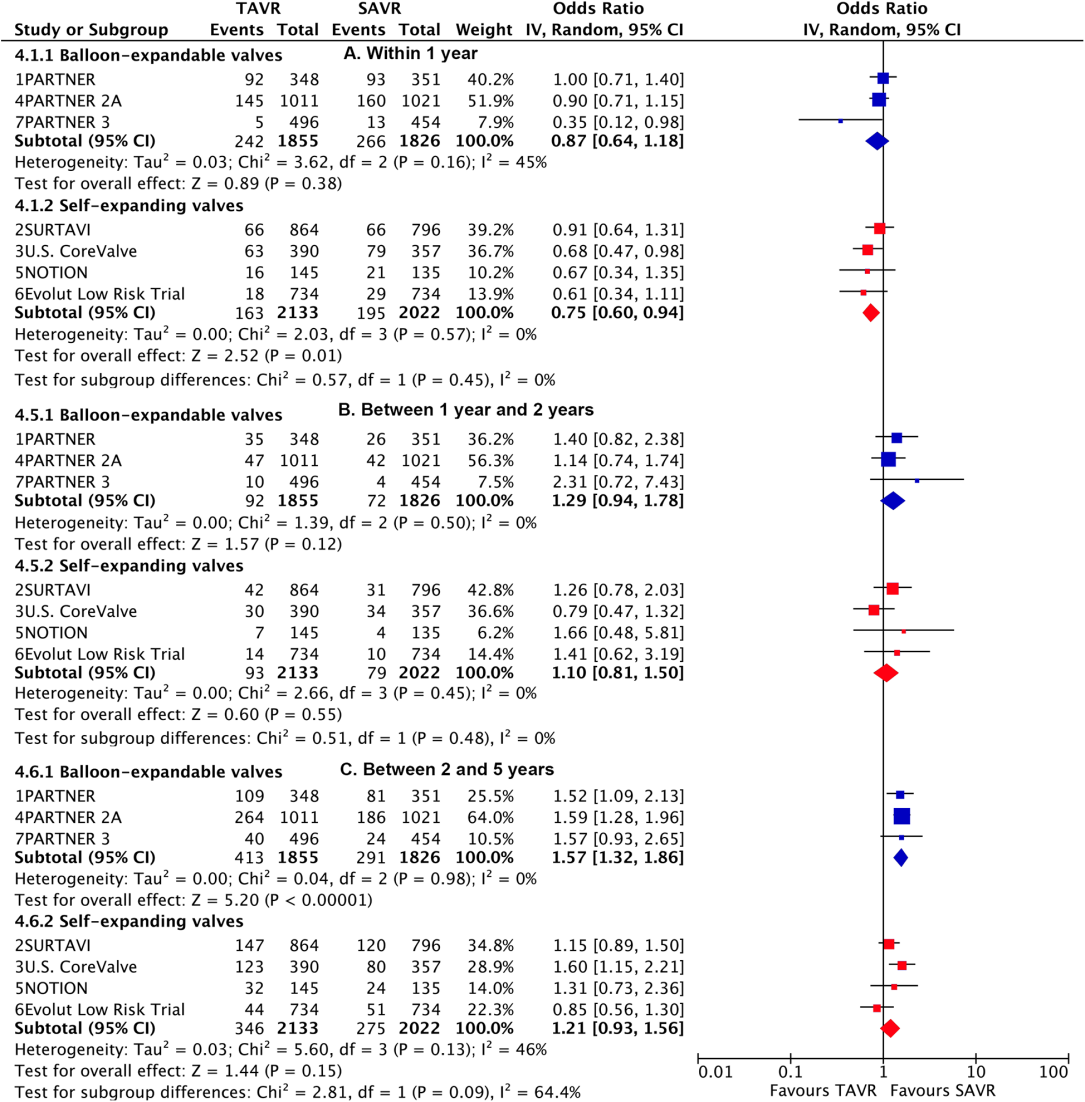
Landmark risk estimates of all-cause death or disabling stroke for TAVI vs SAVR stratified by TAVI valve systems.

Subgroup analysis demonstrated a higher risk of longer-term primary outcome in TAVI using BEV compared to SAVR [1.38 (1.2–1.6)], but no statistical difference was found with SEV [1.03 (0.89–1.19)] (Figure 3). A significant interaction was observed between two valve systems (p for interaction = 0.005, Supplementary Table S8). The higher risk of TAVI with BEV was primarily attributed to events occurring beyond 2 years [1.57 (1.32–1.86)] (p for interaction = 0.004) (Figure 4; Supplementary Table S9). The benefit of TAVI with SEV over SAVR within 1 year [0.75 (0.6–0.94)] was not observed at longer-term follow-up [1.21 (0.93–1.56)], and a significant temporal interaction was detected (p for interaction = 0.015) (Supplementary Table S10).

### 3.3 Other outcomes

Overall and subgroup analysis for all-cause death generated largely similar results with the primary outcome (Supplementary Figures S2, S3). At longer-term follow-up, TAVI was found to have a numerically higher risk of cardiovascular death, a significantly higher risk of TIA, MVC, PPM, reintervention, rehospitalization, and moderate to severe PVL, compared to SAVR. However, TAVI showed a significantly lower risk of major bleeding and new-onset atrial fibrillation, and a comparable risk of stroke and myocardial infarction (Table 1).

**Table 1:** Outcomes at different durations of follow-up for TAVI compared to SAVR.

Outcome or subgroup	Trials	TAVI	SAVR	OR (95% CI)	*P* value
All-cause death					
30-day	8	100/4,446	116/4,303	0.83 (0.61, 1.12)	0.23
1-year	8	366/4,446	401/4,303	0.88 (0.75, 1.02)	0.09
2-year	7	514/3,988	522/3,848	0.95 (0.83, 1.08)	0.43
Longer-term	7	1,268/3,988	1,101/3,848	1.18 (1.03, 1.36)	0.02
Cardiovascular death					
30-day	8	89/4,446	93/4,303	0.93 (0.69, 1.25)	0.633
1-year	8	233/4,446	257/4,303	0.87 (0.73, 1.05)	0.151
2-year	7	320/3,988	329/3,848	0.94 (0.79, 1.1)	0.427
Longer-term	7	755/3,988	675/3,848	1.11 (0.99, 1.26)	0.078
Myocardial infarction					
30-day	8	42/4,446	55/4,303	0.72 (0.48, 1.09)	0.122
1-year	8	78/4,446	83/4,303	0.91 (0.66, 1.25)	0.556
2-year	7	94/3,988	96/3,848	0.96 (0.72, 1.29)	0.805
Longer-term	7	198/3,988	157/3,848	1.12 (0.79, 1.59)	0.514
Stroke					
30-day	8	160/4,446	184/4,303	0.85 (0.62, 1.16)	0.301
1-year	8	240/4,446	246/4,303	0.96 (0.7, 1.3)	0.777
2-year	7	265/3,988	280/3,848	0.9 (0.7, 1.15)	0.407
Longer-term	6	339/3,254	325/3,114	0.99 (0.84, 1.18)	0.953
Transient ischemic attack					
30-day	7	34/3,988	23/3,848	1.45 (0.83, 2.52)	0.190
1-year	7	84/3,988	60/3,848	1.35 (0.97, 1.89)	0.078
2-year	6	99/3,254	64/3,114	1.49 (1.08, 2.06)	0.014
Longer-term	5	128/2,758	94/2,660	1.32 (1, 1.73)	0.046
Major bleeding					
30-day	8	427/4,446	980/4,303	0.35 (0.18, 0.69)	0.003
1-year	6	408/3,437	944/3,372	0.36 (0.23, 0.56)	<0.0001
2-year	4	384/2,483	769/2,463	0.46 (0.25, 0.84)	0.012
Longer-term	2	207/738	247/708	0.71 (0.57, 0.89)	0.003
Major vascular complications					
30-day	8	286/4,446	118/4,303	2.74 (1.74, 4.31)	<0.0001
1-year	6	236/3,437	115/3,372	2.31 (1.48, 3.6)	<0.0001
2-year	4	181/2,483	101/2,463	2.03 (1.2, 3.41)	0.008
Longer-term	2	68/738	21/708	3.39 (2.05, 5.6)	<0.0001
Permanent pacemaker implantation					
30-day	8	652/4,446	248/4,303	2.66 (1.64, 4.31)	<0.0001
1-year	7	495/3,582	244/3,507	2.29 (1.42, 3.7)	0.001
2-year	7	746/3,988	319/3,848	2.57 (1.54, 4.27)	<0.0001
Longer-term	7	852/3,988	395/3,848	2.37 (1.53, 3.68)	<0.0001
New-onset atrial fibrillation					
30-day	7	381/3,988	1,236/3,848	0.22 (0.16, 0.3)	<0.0001
1-year	6	332/3,124	936/3,052	0.27 (0.18, 0.41)	<0.0001
2-year	4	246/2,042	627/1,967	0.26 (0.16, 0.41)	<0.0001
Longer-term	4	330/2,386	804/2,344	0.28 (0.2, 0.38)	<0.0001
Moderate to severe paravalvular leak					
30-day	7	166/3,438	14/3,465	11.4 (6.69, 19.5)	<0.0001
1-year	7	115/3,040	15/2,494	5.67 (3.25, 9.88)	<0.0001
2-year	6	127/1,976	16/1,707	7.97 (2.21, 28.8)	0.002
Longer-term	6	49/1,695	3/1,449	7.9 (3.12, 20.22)	<0.0001
Reintervention					
30-day	5	22/4,098	6/3,952	2.85 (1.16, 7)	0.022
1-year	6	54/4,098	19/3,952	2.48 (1.45, 4.23)	0.001
2-year	4	47/2,906	14/2,763	2.92 (1.3, 6.55)	0.009
Longer-term	6	82/3,640	42/3,497	1.86 (1.05, 3.28)	0.032
Rehospitalization					
30-day	5	130/3,453	153/3,356	0.78 (0.56, 1.1)	0.157
1-year	6	393/3,843	378/3,713	0.97 (0.74, 1.27)	0.828
2-year	6	528/3,843	450/3,713	1.12 (0.88, 1.43)	0.371
Longer-term	6	825/3,843	658/3,713	1.23 (1.0, 1.5)	0.047

The increased risk of TAVI on cardiovascular death was primarily attributed to events occurring beyond 2 years, rehospitalization attributed to events beyond 1 year, while TIA, MVC, and reintervention were primarily associated with events within 1 year. The benefits of TAVI on major bleeding and new-onset atrial fibrillation were mainly attributed to lower events occurring within 1 year. The risk of PPM at longer-term follow-up was primarily attributed to higher events occurring within 1 year in TAVI, with the risk attenuating but still higher in TAVI between 1 year and 2 years and beyond 2 years (Supplementary Table S5).

In subgroup analysis, a statistically higher risk of longer-term reintervention and rehospitalization was observed in TAVI compared to SAVR among participants at higher risk, while no statistical difference was found in patients at lower risk (Supplementary Table S11). Significant interaction was detected between the two risk groups (both p for interaction <0.0001). The lower risk of rehospitalization in TAVI over SAVR in lower-risk patients within the first year was not observed during longer-term follow-up (Supplementary Table S7). Subgroup analysis indicated a statistically higher risk of longer-term PPM in TAVI compared to SAVR, regardless of participants’ higher or lower risk. The SEV showed a higher risk than the BEV, with a significant difference (p for interaction <0.0001) (Supplementary Table S8).

### 3.4 Heterogeneity, publication bias, and sensitivity analyses

There was minimal heterogeneity observed across trials for both the primary outcomes and all death outcomes across all follow-up durations, as detailed in corresponding figures and tables. Several tests for publication bias were conducted for the primary outcome, and no significant results were found (not shown). However, the assessment of publication bias was limited by the relatively small number of trials, potentially affecting the ability to detect small-study effects. The analysis of primary outcome using the Hartung-Knapp-Sidik-Jonkman variance correction and excluding each trial one time revealed largely similar findings (Supplementary Figures S4–S7).

## 4 Discussion

This present meta-analysis, including comprehensive data from all available trials comparing TAVI with SAVR, with >8,000 patients and longer-term follow-up data from nearly all trials, yields several important conclusions (Central Illustration). First, TAVI was associated with a higher risk of longer-term primary outcome compared to SAVR among participants with higher risk, but not among those with lower risk. However, a significant temporal interaction was detected in both risk profiles. Second, TAVI with BEV was associated with a higher risk of longer-term primary outcome compared to SAVR, whereas no statistical difference was found with SEV. There was a significant interaction between the two valve systems, and a temporal interaction was observed in both TAVI systems. Third, landmark analysis revealed a lower risk of primary outcome in TAVI compared to SAVR within the initial 30 days, comparable between 30 days and 2 years, and a significant higher risk beyond 2 years. Fourth, overall analysis showed that TAVI was associated with a higher longer-term risk of all-cause death, TIA, MVC, PPM, reintervention, rehospitalization, and moderate to severe PVL, a comparable risk of stroke and myocardial infarction, but a lower risk of major bleeding and new-onset atrial fibrillation.

**Central Illustration:**
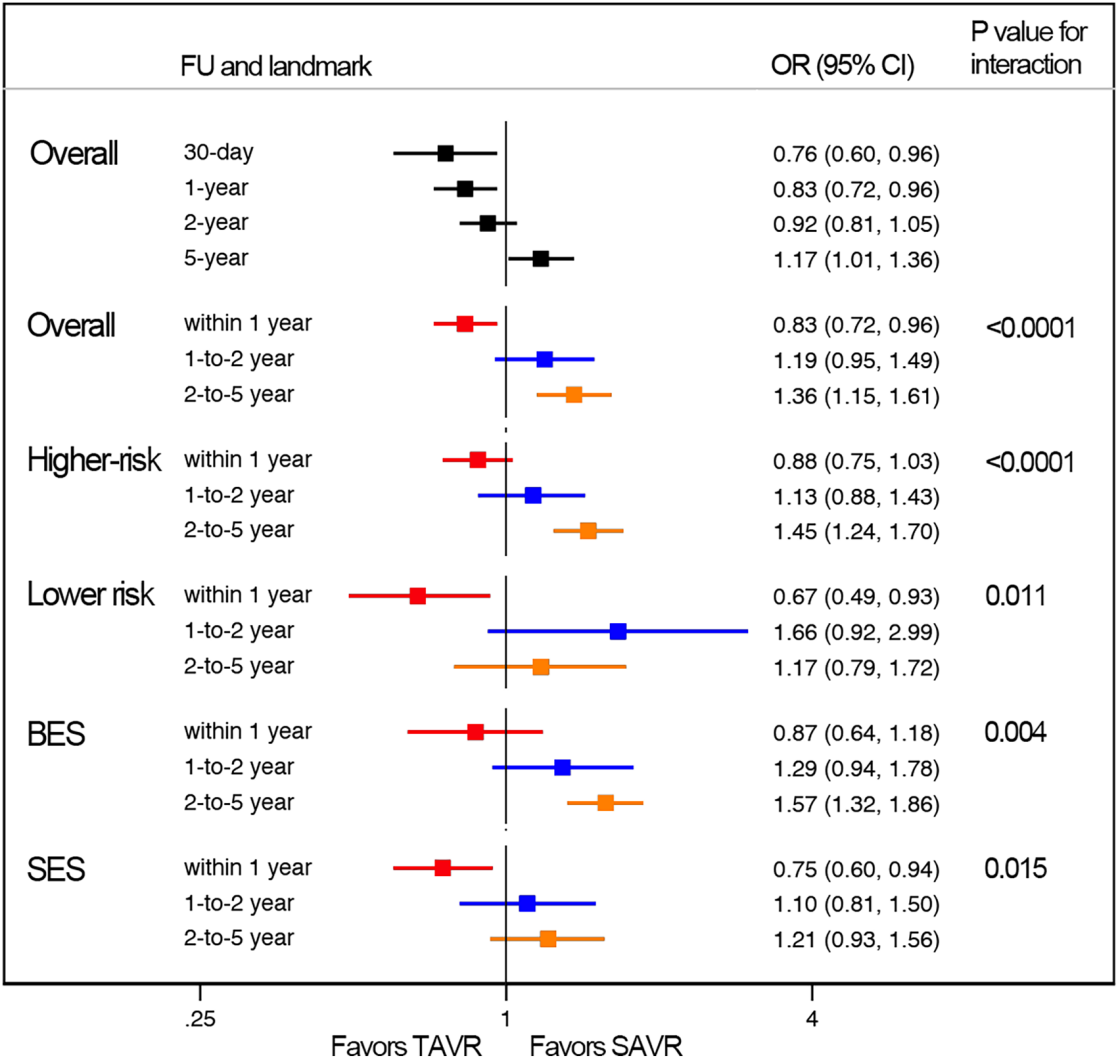
Risk estimates of all-cause death or disabling stroke for TAVI vs SAVR.

We conducted a comprehensive search on PubMed to identify relevant meta-analyses comparing the longer-term outcomes of TAVI and SAVR. However, these meta-analyses included 3–4 trials with 5-year follow-up data, focusing exclusively on patients with higher risks (2, 24, 25). In contrast, our meta-analysis incorporated a larger dataset, comprising 7 trials with longer-term follow-up data, encompassing both higher- and lower-risk patients. It is important to note that our study utilized longer-term data from nearly all registered large RCTs. One of the identified meta-analyses employed a network meta-analysis approach but considered 1-to-2-year follow-up as long-term (26). Another meta-analysis included only 3 RCTs but supplemented them with 7 propensity-score matching observational studies, which were limited by inadequate adjustment for important confounding (27). We also performed several additional analyses. Firstly, we conducted a landmark analysis to assess the differences in TAVI outcomes within specific time intervals, revealing significant temporal variations in the effect of TAVI. Secondly, we conducted subgroup analyses based on TAVI systems and surgical risks, revealing noteworthy distinctions between subgroups.

None of the trials included were specifically designed to have sufficient statistical power to detect a significant reduction in all-cause death. However, our meta-analysis revealed a significant higher risk of longer-term mortality associated with TAVI. This finding aligns with the temporal trend observed in primary outcome. Further subgroup analysis indicated a significantly higher risk of all-cause death in TAVI among higher-risk patients and with BEV, but no significant difference was observed in lower-risk patients or with SEV. Importantly, the temporal trend was also only evident in the former two subgroups. A separate meta-analysis of 7 propensity matched studies corroborated our findings by showing a significantly higher risk of mortality at 5-year follow-up (27).

TAVI demonstrated initial superiority over SAVR within the first year but lost this advantage thereafter in lower risk patients. Given that lower risk patients typically have good life expectancy, this temporal interaction warrants intensive and close attention. In the PARTNER 3 trial, Kaplan–Meier event curves for the primary outcome crossed around the 2- to 3-year mark, thereafter favoring SAVR, while in the Evolut Low Risk trial, the curves remained parallel, favoring TAVI (8, 9). Although there were some differences, the pooled analysis of longer-term data from these lower-risk trials did not show substantial heterogeneity (*I*^2^ = 20%). A large real-world registry including 42,586 patients who underwent isolated SAVR and meeting the inclusion and exclusion criteria for the PARTNER 3 and Evolut Low Risk trials, revealed excellent survival rates in low-risk patients following SAVR, with all-cause mortality of 7.1% at 5 years and 12.4% at 8 years (28). Similar findings were observed in other large registries (29). Determining whether TAVI can achieve such excellent long-term outcomes as SAVR will require robust evidence from follow-up periods exceeding 10 years. The recommendation of TAVI in these patients is pending this evidence.

We showed a higher longer-term risk of primary outcome and all-cause death in TAVI compared to SAVR among higher-risk patients. These observations seem a paradox, i.e., patients with a higher surgical risk actually had better longer-term outcomes when they underwent surgery instead of opting for TAVI. Notably, the short-term risk of all-cause death was not decreased in TAVI in higher risk patients. This observation was similar to several meta-analyses with higher-risk patients (2, 24). Unfortunately, no randomized trials in high-risk patients using newer-generation valves have been conducted thus far. There have been some propensity-matched studies that shed light on this topic. For instance, a study involving 72 pairs of high-risk patients, although utilizing mixed generations of TAVI valves, showed a lower in-hospital mortality rate but a higher risk of all-cause death at 5-year follow-up in the TAVI group (30). Another propensity-matched analysis of 783 pairs of intermediate-risk patients (mean age: 81.7 years, mean STS score: 5.5) using newer-generation SAPIEN 3 valves demonstrated a comparable risk of death or disabling stroke at 5 years compared to SAVR (31). Further studies are warranted to evaluate the performance of TAVI with newer-generation valves compared to SAVR in the context of higher-risk patients.

An interesting finding of our analysis was the significant interaction between BEV and SEV regarding the primary outcome and all-cause death at longer-term follow-up. A temporal interaction was observed in BEV for both the primary outcome and all-cause death, while in SEV, it was observed only for the primary outcome. These temporal trends closely align with those reported in the PARTNER 2A trial (5), which compared early-generation BEV TAVI with SAVR in higher surgical risk patients, and the PARTNER 3 trial (9), which compared newer-generation BEV TAVI with SAVR in lower surgical risk patients. Landmark analyses of clinical events between 2 and 5 years in both trials demonstrated higher rates of all-cause death and the primary outcome in TAVI compared to SAVR. Similarly, in another trial of BEV TAVI, the Kaplan–Meier event curves for all-cause death converged at 2 years (4). In contrast, trials comparing SEV TAVI to SAVR showed Kaplan–Meier event curves for the primary endpoint that remained parallel, favoring TAVI in the Evolut Low Risk trial (8), nearly overlapped in the SURTAVI (6) and NOTION (20) trials, and converged until the 5-year mark in the U.S. CoreValve trial (3). Longer-term data from head-to-head comparisons of BEV with SEV TAVI have been reported in only one RCT (32). In this trial, with 241 high-risk patients randomly assigned to early generation BEV and SEV, all-cause mortality (53.4% vs. 47.6%) and cardiovascular mortality (31.6% vs. 21.5%) at 5 years were numerically higher in the BEV group compared with the SEV group, consistent with our findings. These differences might be attributed to better forward flow hemodynamics and less structural valve deterioration in SEV compared to BEV (32). Several propensity-matched studies showed varied findings, but these conclusions were limited by residual confounders that could not be fully accounted for, such as patients’ anatomical suitability. It is likely that more patients with extensive outflow tract calcifications, low implanted coronary arteries, or complex and small femoral access received SEV (33). We found no significant difference between BEV and SEV at short-term follow-up, which is also consistent with findings from other RCTs (34, 35).

Our analysis had several strengths. Firstly, we incorporated the largest number of RCTs with longer-term follow-up outcomes, ensuring a comprehensive evaluation of the data. Additionally, the trials included in our analysis had nearly identical follow-up durations, enabling landmark analyses and mitigating the potential impact of variations in follow-up durations on the outcomes.

However, it is important to acknowledge some limitations. Firstly, our analysis was based on trial-level rather than patient-level data. Although we performed subgroup analyses based on clinically relevant subgroups, we were unable to conduct more detailed meta-regression analyses to account for potential confounding factors beyond the subgroup variables. Secondly, concomitant procedures were performed in both TAVI and SAVR groups in original trials, which could potentially influence the evaluation of isolated TAVI vs. isolated SAVR. Thirdly, our assessment of publication bias was limited by the relatively small number of trials, potentially affecting the ability to detect small-study effects.

## 5 Conclusions

TAVI was associated with a higher longer-term risk of primary outcome compared to SAVR in higher-risk patients and with balloon-expandable valves. However, a characteristic temporal interaction was documented in all subgroups. Long-term follow-up data from low-risk trials and large trials comparing TAVI with balloon-expandable and self-expanding valves are warranted to test these findings.

## Data Availability

The original contributions presented in the study are included in the article/Supplementary Material, further inquiries can be directed to the corresponding authors.
