# Supplementary material for: Longer-term and landmark analysis of transcatheter vs. surgical aortic-valve implantation in severe aortic stenosis: a meta-analysis
Source: Front Cardiovasc Med. 2025 Mar 6;12:1479200. doi: 10.3389/fcvm.2025.1479200 (PMC11922901; doi:10.3389/fcvm.2025.1479200)
Supplement: Supplementary file 1 [file Datasheet1.docx]

**SUPPLEMENTARY MATERIALS**

**Longer-term and Landmark Analysis of Transcatheter versus Surgical Aortic-Valve Implantation in Severe Aortic Stenosis: A Meta-Analysis**

Yu Wang, MS; Xiaowen Zhang, MD; Xinlin Zhang, MD; Wei Xu, MD

**Search strategy for PubMed:**

1. transcatheter aortic valve implantation[Title/Abstract]
2. transcatheter aortic valve replacement[Title/Abstract]
3. TAVI[Title/Abstract]
4. TAVR[Title/Abstract]
5. 1 or 2 or 3 or 4
6. randomised controlled trial[Title/Abstract]
7. controlled trial[Title/Abstract]
8. random*[Title/Abstract]
9. 6 or 7 or 8
10. 5 and 9

**Table S1.** PRISMA checklist

| **Section and Topic** | **Item #** | **Checklist item** | **Reported？** |
| --- | --- | --- | --- |
| **TITLE** | | |  |
| Title | 1 | Identify the report as a systematic review. | Yes |
| **ABSTRACT** | | |  |
| Abstract | 2 | See the PRISMA 2020 for Abstracts checklist. | Yes |
| **INTRODUCTION** | | |  |
| Rationale | 3 | Describe the rationale for the review in the context of existing knowledge. | Yes |
| Objectives | 4 | Provide an explicit statement of the objective(s) or question(s) the review addresses. | Yes |
| **METHODS** | | |  |
| Eligibility criteria | 5 | Specify the inclusion and exclusion criteria for the review and how studies were grouped for the syntheses. | Yes |
| Information sources | 6 | Specify all databases, registers, websites, organisations, reference lists and other sources searched or consulted to identify studies. Specify the date when each source was last searched or consulted. | Yes |
| Search strategy | 7 | Present the full search strategies for all databases, registers and websites, including any filters and limits used. | Yes |
| Selection process | 8 | Specify the methods used to decide whether a study met the inclusion criteria of the review, including how many reviewers screened each record and each report retrieved, whether they worked independently, and if applicable, details of automation tools used in the process. | Yes |
| Data collection process | 9 | Specify the methods used to collect data from reports, including how many reviewers collected data from each report, whether they worked independently, any processes for obtaining or confirming data from study investigators, and if applicable, details of automation tools used in the process. | Yes |
| Data items | 10a | List and define all outcomes for which data were sought. Specify whether all results that were compatible with each outcome domain in each study were sought (e.g. for all measures, time points, analyses), and if not, the methods used to decide which results to collect. | Yes |
|  | 10b | List and define all other variables for which data were sought (e.g. participant and intervention characteristics, funding sources). Describe any assumptions made about any missing or unclear information. | Yes |
| Study risk of bias assessment | 11 | Specify the methods used to assess risk of bias in the included studies, including details of the tool(s) used, how many reviewers assessed each study and whether they worked independently, and if applicable, details of automation tools used in the process. | Yes |
| Effect measures | 12 | Specify for each outcome the effect measure(s) (e.g. risk ratio, mean difference) used in the synthesis or presentation of results. | Yes |
| Synthesis methods | 13a | Describe the processes used to decide which studies were eligible for each synthesis (e.g. tabulating the study intervention characteristics and comparing against the planned groups for each synthesis (item #5)). | Yes |
|  | 13b | Describe any methods required to prepare the data for presentation or synthesis, such as handling of missing summary statistics, or data conversions. | Yes |
|  | 13c | Describe any methods used to tabulate or visually display results of individual studies and syntheses. | Yes |
|  | 13d | Describe any methods used to synthesize results and provide a rationale for the choice(s). If meta-analysis was performed, describe the model(s), method(s) to identify the presence and extent of statistical heterogeneity, and software package(s) used. | Yes |
|  | 13e | Describe any methods used to explore possible causes of heterogeneity among study results (e.g. subgroup analysis, meta-regression). | Yes |
|  | 13f | Describe any sensitivity analyses conducted to assess robustness of the synthesized results. | Yes |
| Reporting bias assessment | 14 | Describe any methods used to assess risk of bias due to missing results in a synthesis (arising from reporting biases). | Yes |
| Certainty assessment | 15 | Describe any methods used to assess certainty (or confidence) in the body of evidence for an outcome. | - |
| **RESULTS** | | |  |
| Study selection | 16a | Describe the results of the search and selection process, from the number of records identified in the search to the number of studies included in the review, ideally using a flow diagram. | Yes |
|  | 16b | Cite studies that might appear to meet the inclusion criteria, but which were excluded, and explain why they were excluded. | - |
| Study characteristics | 17 | Cite each included study and present its characteristics. | Yes |
| Risk of bias in studies | 18 | Present assessments of risk of bias for each included study. | Yes |
| Results of individual studies | 19 | For all outcomes, present, for each study: (a) summary statistics for each group (where appropriate) and (b) an effect estimate and its precision (e.g. confidence/credible interval), ideally using structured tables or plots. | Yes |
| Results of syntheses | 20a | For each synthesis, briefly summarise the characteristics and risk of bias among contributing studies. | Yes |
|  | 20b | Present results of all statistical syntheses conducted. If meta-analysis was done, present for each the summary estimate and its precision (e.g. confidence/credible interval) and measures of statistical heterogeneity. If comparing groups, describe the direction of the effect. | Yes |
|  | 20c | Present results of all investigations of possible causes of heterogeneity among study results. | Yes |
|  | 20d | Present results of all sensitivity analyses conducted to assess the robustness of the synthesized results. | Yes |
| Reporting biases | 21 | Present assessments of risk of bias due to missing results (arising from reporting biases) for each synthesis assessed. | Yes |
| Certainty of evidence | 22 | Present assessments of certainty (or confidence) in the body of evidence for each outcome assessed. | - |
| **DISCUSSION** | | |  |
| Discussion | 23a | Provide a general interpretation of the results in the context of other evidence. | Yes |
|  | 23b | Discuss any limitations of the evidence included in the review. | Yes |
|  | 23c | Discuss any limitations of the review processes used. | Yes |
|  | 23d | Discuss implications of the results for practice, policy, and future research. | Yes |
| **OTHER INFORMATION** | | |  |
| Registration and protocol | 24a | Provide registration information for the review, including register name and registration number, or state that the review was not registered. | - |
|  | 24b | Indicate where the review protocol can be accessed, or state that a protocol was not prepared. | - |
|  | 24c | Describe and explain any amendments to information provided at registration or in the protocol. | - |
| Support | 25 | Describe sources of financial or non-financial support for the review, and the role of the funders or sponsors in the review. | Yes |
| Competing interests | 26 | Declare any competing interests of review authors. | Yes |
| Availability of data, code and other materials | 27 | Report which of the following are publicly available and where they can be found: template data collection forms; data extracted from included studies; data used for all analyses; analytic code; any other materials used in the review. | - |

**Table S2.** Baseline characteristics of included randomized controlled trials

| **Trial** | **PARTNER** | **U.S. CoreValve** | **NOTION** | **PARTNER 2A** | **SURTAVI** | **PARTNER 3** | **Evolut Low Risk** | **UK TAVI** |
| --- | --- | --- | --- | --- | --- | --- | --- | --- |
| Year of first publication | 2011 | 2014 | 2015 | 2016 | 2017 | 2019 | 2019 | 2023 |
| Year of last publication | 2015 | 2018 | 2021 | 2020 | 2022 | 2023 | 2023 | 2023 |
| Recruitment period | 2007-2009 | 2011-2012 | 2009-2013 | 2011-2013 | 2012-2016 | 2016-2017 | 2016-2018 | 2012-2017 |
| Number of centers | 25 | 45 | 3 | 57 | 87 | 71 | 86 | 31 |
| No. of patients | 699 | 795 | 280 | 2,032 | 1,746 | 1,000 | 1,468 | 913 |
| Available follow-up, months | 1, 12, 24, 60 | 1, 12, 24, 36, 60 | 1, 12, 24, 60, 96 | 1, 12, 24, 24, 60 | 1, 12, 24, 24, 60 | 1, 12, 24, 60 | 1, 12, 24, 48 | 1, 12 |
| Age, ys | 83.6±6.8 | 83.2±7.1 | 79.2±4.9 | 81.5±6.7 | 79.8±6.2 | 73.3±5.8 | 74.0±5.9 | 81 (79, 84) |
| Male, % | 57.1 | 43.2 | 53.2 | 54.5 | 56.8 | 69.2 | 65.1 | 53.6 |
| STS-PROM score, % | 11.8±3.3 | 7.3±3.0 | 2.9±1.6 | 5.8±2.1 | 4.5±1.6 | 1.9±0.7 | 1.9±0.7 | 2.6 (2.0, 3.5) |
| Logistic EuroSCORE | 29.3±16.5 | 17.6±13.0 | 8.4±4.0 | NA | 11.9±7.6 | 1.5±1.2 | NA | 2.0 (1.4, 3.0) |
| Risk | Higher risk | Higher risk | Lower risk | Higher risk | Higher risk | Lower risk | Lower risk | Lower risk |
| Aortic-valve gradient, mmHg | 42.7±14.6 | NA | NA | 44.9±13.4 | NA | 49.4±12.8 | 47.2±12.3 | 73 (59-89) |
| Aortic-valve area, cm^2^ | 0.7±0.2 | NA | NA | 0.7±0.2 | NA | 0.8±0.2 | 0.8±0.2 | 0.7 (0.6, 0.9) |
| Moderate or severe mitral regurgitation, % | 20.5 | NA | NA | 17.9 | NA | 3.2 | NA | 12.0 |
| TAVI valve system | Edwards SAPIEN | Medtronic CoreValve | Medtronic CoreValve | Edwards SAPIEN XT | Medtronic CoreValve (84%), Evolut R (16%) | Edwards SAPIEN 3 | Medtronic CoreValve (3.6%), Evolut R (74.1%), Evolut PRO (22.3%) | SAPIEN 3 (45.1%), Evolut R (13.6%), Lotus (9.6%), CoreValve (8.4%), SAPIEN (8.0%), others (15.3%) |
| Transfemoral, % | 70.1 | 82.8 | 96.5 | 76.3 | 93.6 | 100 | 99 | 92.0 |
| Diabetes, % | NA | 26.2 | 19.3 | 35.9 | 34.5 | 20.7 | 30.8 | 24.0 |
| Hypertension, % | NA | 95.7 | 73.6 | NA | 91.8 | NA | 83.8 | 72.2 |
| Coronary artery disease, % | 75.9 | 75.8 | NA | 67.9 | 63.3 | 27.8 | NA | 31.6 |
| Prior PCI, % | 33.2 | 35.8 | 8.2 | 27.4 | 21.1 | NA | 13.3 | 10.6 |
| Prior CABG, % | 43.4 | 29.9 | NA | 19.7 | 16.4 | NA | 2.4 | 2.0 |
| Prior MI, % | 27.9 | 25 | 5 | 17.9 | 13.8 | 5.7 | 6 | 9.3 |
| PAD, % | 42.3 | 42 | 5.4 | 30.4 | 30.5 | 7.1 | 8 | NA |
| Prior stroke/TIA, % | 28.4 | 12.7 | 16.4 | 31.6 | 10.6 | 4.2 | 10.8 | 6.3 |
| Permanent pacemaker, % | 21 | 21.4 | 3.9 | 11.9 | 9.3 | 2.6 | 3.6 | 6.6 |
| Prior AF/atrial flutter, % | 41.8 | 44.2 | 26.4 | 33.1 | 27.3 | 17.2 | 15.2 | 24.1 |
| Creatinine >2 mg/dl, % | 9.1 | 12.7 | 1.1 | 5.1 | 2 | 0.2 | 0.3 | NA |
| COPD, % | 43.2 | NA | 11.8 | 30.9 | 34.5 | 5.6 | 15.5 | 22.1 |

AF: atrial fibrillation; CABG: coronary-artery bypass grafting; COPD: chronic obstructive pulmonary disease; EuroSCORE: Scores on the European System for Cardiac Operative Risk Evaluation; MI: myocardial infarction; NA: not available; PAD: peripheral vascular disease; PCI: percutaneous coronary intervention; STS-PROM: Society of Thoracic Surgeons Predicted Risk Of Mortality; TAVI: transcatheter aortic-valve implantation; TIA: transient ischemic attack.

**Table S3.** Main inclusion criteria, primary and secondary endpoints of randomized controlled trials.

| **Trial** | **Main inclusion criteria** | **Primary endpoint** | **Main secondary endpoint** |
| --- | --- | --- | --- |
| PARTNER | Severe aortic stenosis (AS) and cardiac symptoms (NYHA ≥ II), with aortic-valve area [AVA] ≤ 0.8 cm2 plus either mean valve gradient ≥ 40 mm Hg or a peak velocity ≥4.0 m/s, with high risk for surgery (risk of 30-day death of ≥15%) | Death from any cause at 1 year | Death from cardiovascular causes, NYHA functional class, repeat hospitalization because of valve- or procedure-related clinical deterioration, myocardial infarction, stroke, acute kidney injury, vascular complications, bleeding, 6-minute walk distance, and valve performance. |
| U.S. CoreValve | Severe AS and heart failure symptoms (NYHA ≥ II), with AVA ≤0.8 cm2 or an aortic-valve index ≤0.5 cm2/m2 and either a mean aortic-valve gradient ≥ 40 mm Hg or a peak aortic-jet velocity ≥ 4.0 m/s, with high risk for surgery (the risk of 30-day death ≥15% and the risk of death or 30-day irreversible complications <50%. | Death from any cause at 1 year | Composite of major adverse cardiovascular and cerebrovascular events (defined as a composite of death from any cause, myocardial infarction, any stroke, or reintervention) at 30 days and 1 year, as well as the individual components of this composite. |
| NOTION | Severe AS >70 years of age and cardiac symptoms (NYHA ≥ II), with AVA ≤1.0 cm2 or an aortic-valve index ≤0.6 cm2/m2 and either a mean aortic-valve gradient > 40 mm Hg or a peak systolic velocity > 4.0 m/s, without surgical risk restriction. | Composite rate of all-cause death, stroke, or myocardial infarction at 1 year | The rate of individual components of the composite outcome; the rate of cardiovascular death; prosthesis reintervention; cardiogenic shock; valve endocarditis; conduction abnormalities requiring permanent pacemaker; atrial fibrillation or flutter; and vascular, renal, and bleeding complications after 1 and 12 months. |
| PARTNER 2A | Severe AS and cardiac symptoms (NYHA ≥ II), with AVA ≤0.8 cm2 or an aortic-valve index ≤0.5 cm2/m2, or either a mean aortic-valve gradient >40 mm Hg or a peak systolic velocity >4.0 m/s, intermediate surgical risk (STS score 4-8%, or <4% but deemed as intermediate risk if with coexisting conditions that were not represented in the risk model) | Composite of death from any cause or disabling stroke at 2 years | Death from cardiovascular causes, repeat hospitalization because of valve- or procedure-related clinical deterioration, myocardial infarction, stroke, acute kidney injury, vascular complications, bleeding, 6-minute walk distance, and valve performance. |
| SURTAVI | Severe AS and cardiac symptoms (NYHA ≥ II), with AVA ≤1.0 cm2 or an aortic-valve index ≤0.6 cm2/m2, or either a mean aortic-valve gradient >40 mm Hg or a peak systolic velocity >4.0 m/s, intermediate surgical risk (STS-PROM 30-day surgical death 3-15%) | All-cause death or disabling stroke at 2 years | Major adverse cardiovascular and cerebrovascular events, which consisted of death from any cause, myocardial infarction, all types of strokes, and any reintervention. |
| Evolut Low Risk | Severe AS and cardiac symptoms, with AVA ≤1.0 cm2 or an aortic-valve index ≤0.6 cm2/m2, or either a mean aortic-valve gradient > 40 mm Hg or a peak systolic velocity > 4.0 m/s, low surgical risk (STS-PROM 30-day surgical death <3%) | A composite of death from any cause or disabling stroke at 2 years | A composite of death, disabling stroke, life-threatening bleeding, major vascular complication, or stage 2 or 3 acute kidney injury at 30 days; and prosthetic-valve endocarditis, prosthetic-valve thrombosis, prosthetic-valve dysfunction requiring a repeat procedure, stroke, and life-threatening bleeding at 12 months. |
| PARTNER 3 | Severe AS and cardiac symptoms (NYHA ≥ II), asymptomatic with LVEF <50% or exercise tolerance test abnormity, with AVA ≤1.0 cm2 or an aortic-valve index ≤0.6 cm2/m2, or either a mean aortic-valve gradient ≥40 mm Hg or a peak systolic velocity ≥4.0 m/s, low surgical risk (STS-PROM 30-day surgical death <4%) | Composite of death from any cause, stroke, or rehospitalization at 1 year | Stroke, a composite of death or stroke, and new-onset atrial fibrillation at 30 days, as well as the length of the index hospitalization and a poor treatment outcome, which was a composite of death or a low Kansas City Cardiomyopathy Questionnaire (KCCQ) overall summary score (with scores ranging from 0 to 100 and higher scores indicating fewer physical limitations and a greater feeling of well-being) at 30 days. |
| UK TAVI | Severe, symptomatic AS aged >70 years with increased operative risk due to comorbidity, or with age ≥80 years. | Death from any cause at 1 year | Cardiovascular death; stroke; reintervention; a composite of death or stroke; a composite of death or disabling stroke; a composite of death, disabling stroke, or reintervention; vascular complications; major bleeding events; conduction disturbances requiring permanent pacing; myocardial infarction; kidney replacement therapy; and infective endocarditis. |

**Table S4.** Risk of bias of included randomized controlled trials.

| **Trial** | **Year** | **Randomization process** | **Deviations from intended interventions** | **Missing outcome data** | **Measurement of the outcome** | **Selection of the reported result** | **Overall bias** |
| --- | --- | --- | --- | --- | --- | --- | --- |
| PARTNER | 2011 | Some concerns | Low risk | Low risk | Low risk | Low risk | Low risk |
| U.S. CoreValve | 2014 | Some concerns | Low risk | Low risk | Low risk | Low risk | Low risk |
| NOTION | 2015 | Some concerns | Low risk | Low risk | Low risk | Low risk | Low risk |
| PARTNER 2A | 2016 | Some concerns | Low risk | Low risk | Low risk | Low risk | Low risk |
| SURTAVI | 2017 | Some concerns | Low risk | Low risk | Low risk | Low risk | Low risk |
| Evolut Low Risk Trial | 2019 | Some concerns | Low risk | Low risk | Low risk | Low risk | Low risk |
| PARTNER 3 | 2019 | Some concerns | Low risk | Low risk | Low risk | Low risk | Low risk |
| UK TAVI | 2023 | Some concerns | Low risk | Low risk | Low risk | Low risk | Low risk |

**Table S5.** Landmark analysis of outcomes for TAVI compared with SAVR.

|  | **Studies** | **TAVI** | **SAVR** | **Effect Estimate** | **P value** | **I2, %** | **Subgroup difference** | |
| --- | --- | --- | --- | --- | --- | --- | --- | --- |
|  |  |  |  |  |  |  | **P value** | **X2** |
| **All-cause death or disabling stroke** |  |  |  |  |  |  | 0.000 | 20.5 |
| Within 30 days | 7 | 155/4301 | 198/4168 | 0.76 (0.6, 0.96) | 0.021 | 6.4 |  |  |
| 30 days to 1 year | 8 | 280/4446 | 298/4303 | 0.89 (0.73, 1.09) | 0.261 | 8.5 |  |  |
| 1 year to 2 years | 7 | 185/3988 | 151/3848 | 1.19 (0.95, 1.49) | 0.128 | 4.6 |  |  |
| Beyond 2 years | 7 | 759/3988 | 566/3848 | 1.36 (1.15, 1.61) | 0.000 | 9.8 |  |  |
| **All-cause death** |  |  |  |  |  |  | 0.03 | 8.7 |
| Within 30 days | 8 | 100/4446 | 116/4303 | 0.83 (0.61, 1.12) | 0.226 | 7.7 |  |  |
| 30 days to 1 year | 8 | 266/4446 | 285/4303 | 0.9 (0.74, 1.08) | 0.263 | 7.5 |  |  |
| 1 year to 2 years | 7 | 169/3988 | 151/3848 | 1.08 (0.86, 1.36) | 0.497 | 4.5 |  |  |
| Beyond 2 years | 7 | 589/3988 | 460/3848 | 1.26 (1.05, 1.52) | 0.014 | 10.2 |  |  |
| **Cardiovascular death** |  |  |  |  |  |  | 0.010 | 8.9 |
| Within 1 year | 8 | 233/4446 | 257/4303 | 0.87 (0.73, 1.05) | 0.151 | 5.2 |  |  |
| 1 year to 2 years | 7 | 100/3988 | 87/3848 | 1.1 (0.82, 1.48) | 0.518 | 5.1 |  |  |
| Beyond 2 years | 7 | 435/3988 | 346/3848 | 1.26 (1.08, 1.46) | 0.003 | 3.6 |  |  |
| **Stroke** |  |  |  |  |  |  | 0.440 | 1.7 |
| Within 1 year | 8 | 240/4446 | 246/4303 | 0.96 (0.7, 1.3) | 0.777 | 15.7 |  |  |
| 1 year to 2 years | 7 | 49/3988 | 46/3848 | 1.02 (0.64, 1.6) | 0.947 | 6.8 |  |  |
| Beyond 2 years | 6 | 117/3254 | 86/3114 | 1.29 (0.91, 1.85) | 0.154 | 6.8 |  |  |
| **Transient ischemic attack** |  |  |  |  |  |  | 0.300 | 2.4 |
| Within 1 year | 7 | 84/3988 | 60/3848 | 1.35 (0.97, 1.89) | 0.078 | 1.6 |  |  |
| 1 year to 2 years | 6 | 27/3254 | 16/3114 | 1.6 (0.71, 3.57) | 0.256 | 6.5 |  |  |
| Beyond 2 years | 5 | 34/2758 | 37/2660 | 0.89 (0.56, 1.44) | 0.647 | 2.5 |  |  |
| **Myocardial infarction** |  |  |  |  |  |  | 0.190 | 3.4 |
| Within 1 year | 8 | 78/4446 | 83/4303 | 0.91 (0.66, 1.25) | 0.556 | 3.3 |  |  |
| 1 year to 2 years | 7 | 23/3988 | 18/3848 | 1.25 (0.66, 2.38) | 0.491 | 4.9 |  |  |
| Beyond 2 years | 7 | 104/3988 | 61/3848 | 1.56 (0.94, 2.58) | 0.086 | 10.0 |  |  |
| **Major bleeding** |  |  |  |  |  |  | <0.0001 | 20.3 |
| Within 1 year | 6 | 408/3437 | 944/3372 | 0.36 (0.23, 0.56) | 0.000 | 48.7 |  |  |
| 1 year to 2 years | 4 | 47/2483 | 34/2463 | 1.37 (0.88, 2.15) | 0.165 | 0.8 |  |  |
| Beyond 2 years | 2 | 24/738 | 17/708 | 1.36 (0.65, 2.82) | 0.416 | 1.3 |  |  |
| **Major vascular complications** |  |  |  |  |  |  | 0.670 | 0.8 |
| Within 1 year | 6 | 236/3437 | 115/3372 | 2.31 (1.48, 3.6) | 0.000 | 15.1 |  |  |
| 1 year to 2 years | 4 | 6/2483 | 4/2463 | 1.36 (0.34, 5.4) | 0.664 | 2.7 |  |  |
| Beyond 2 years | 1 | 1/348 | 1/351 | 1.01 (0.06, 16.19) | 0.995 | 0.0 |  |  |
| **Permanent pacemaker implantation** |  |  |  |  |  |  | 0.150 | 3.8 |
| Within 1 year | 7 | 495/3582 | 244/3507 | 2.29 (1.42, 3.7) | 0.001 | 43.5 |  |  |
| 1 year to 2 years | 6 | 57/3124 | 39/3052 | 1.41 (0.93, 2.14) | 0.101 | 1.7 |  |  |
| Beyond 2 years | 7 | 106/3988 | 76/3848 | 1.31 (0.96, 1.78) | 0.089 | 6.1 |  |  |
| **New-onset atrial fibrillation** |  |  |  |  |  |  | <0.0001 | 29.0 |
| Within 1 year | 6 | 332/3124 | 936/3052 | 0.27 (0.18, 0.41) | 0.000 | 41.1 |  |  |
| 1 year to 2 years | 4 | 27/2042 | 11/1967 | 1.99 (0.95, 4.16) | 0.067 | 2.9 |  |  |
| Beyond 2 years | 3 | 55/1652 | 22/1610 | 2.63 (0.75, 9.19) | 0.129 | 5.7 |  |  |
| **Reintervention** |  |  |  |  |  |  | 0.090 | 4.8 |
| Within 1 year | 6 | 54/4098 | 19/3952 | 2.48 (1.45, 4.23) | 0.001 | 3.9 |  |  |
| 1 year to 2 years | 4 | 8/2906 | 4/2763 | 1.62 (0.46, 5.68) | 0.454 | 1.9 |  |  |
| Beyond 2 years | 6 | 35/3640 | 28/3497 | 1.07 (0.63, 1.81) | 0.795 | 4.9 |  |  |
| **Rehospitalization** |  |  |  |  |  |  | 0.007 | 9.8 |
| Within 1 year | 6 | 393/3843 | 378/3713 | 0.97 (0.74, 1.27) | 0.828 | 14.4 |  |  |
| 1 year to 2 years | 6 | 135/3843 | 72/3713 | 1.83 (1.36, 2.46) | 0.000 | 5.1 |  |  |
| Beyond 2 years | 6 | 297/3843 | 208/3713 | 1.39 (1.11, 1.75) | 0.005 | 7.2 |  |  |

**Table S6.** Landmark analysis of outcomes for TAVI compared with SAVR in participants with higher risk.

|  | **Studies** | **TAVI** | **SAVR** | **Effect Estimate** | **P value** | **I2, %** | **Subgroup difference** | |
| --- | --- | --- | --- | --- | --- | --- | --- | --- |
|  |  |  |  |  |  |  | **P value** | **x2** |
| **All-cause death or disabling stroke** |  |  |  |  |  |  |  |  |
| Within 1 year | 4 | 366/2613 | 398/2525 | 0.88 (0.75, 1.03) | 0.101 | 0 | <0.0001 | 19.5 |
| 1 year to 2 years | 4 | 154/2613 | 133/2525 | 1.13 (0.88, 1.43) | 0.337 | 0 |  |  |
| Beyond 2 years | 4 | 643/2613 | 467/1021 | 1.45 (1.24, 1.7) | <0.0001 | 24.4 |  |  |
| **All-cause death** |  |  |  |  |  |  |  |  |
| Within 1 year | 4 | 318/2613 | 332/2525 | 0.92 (0.78, 1.09) | 0.353 | 0 | 0.001 | 14.2 |
| 1 year to 2 years | 4 | 147/2613 | 131/2525 | 1.09 (0.85, 1.39) | 0.503 | 0 |  |  |
| Beyond 2 years | 4 | 651/2613 | 489/2525 | 1.39 (1.22, 1.6) | <0.0001 | 5.1 |  |  |
| **Cardiovascular death** |  |  |  |  |  |  |  |  |
| Within 1 year | 4 | 198/2613 | 205/2525 | 0.93 (0.76, 1.14) | 0.494 | 0 | 0.062 | 5.55 |
| 1 year to 2 years | 4 | 89/2613 | 76/2525 | 1.14 (0.77, 1.68) | 0.504 | 32.2 |  |  |
| Beyond 2 years | 4 | 375/2613 | 296/2525 | 1.28 (1.08, 1.51) | 0.004 | 0 |  |  |
| **Stroke** |  |  |  |  |  |  |  |  |
| Within 1 year | 4 | 175/2613 | 185/2525 | 0.93 (0.65, 1.32) | 0.682 | 56.6 | 0.666 | 0.81 |
| 1 year to 2 years | 4 | 30/2613 | 31/2525 | 0.91 (0.46, 1.83) | 0.796 | 41.4 |  |  |
| Beyond 2 years | 4 | 94/2613 | 72/2525 | 1.2 (0.75, 1.93) | 0.447 | 48.7 |  |  |
| **Permanent pacemaker implantation** |  |  |  |  |  |  |  |  |
| Within 1 year | 3 | 202/1749 | 139/1729 | 1.51 (0.93, 2.45) | 0.093 | 72 | 0.58 | 1.09 |
| 1 year to 2 years | 3 | 31/1749 | 18/1729 | 1.68 (0.93, 3.04) | 0.084 | 0 |  |  |
| Beyond 2 years | 4 | 68/2613 | 54/2525 | 1.21 (0.84, 1.74) | 0.306 | 0 |  |  |
| **Rehospitalization** |  |  |  |  |  |  |  |  |
| Within 1 year | 4 | 334/2613 | 285/2525 | 1.16 (0.98, 1.38) | 0.081 | 0 | 0.04 | 6.41 |
| 1 year to 2 years | 4 | 113/2613 | 58/2525 | 1.9 (1.33, 2.72) | <0.0001 | 15.3 |  |  |
| Beyond 2 years | 4 | 242/2613 | 166/2525 | 1.44 (1.04, 1.98) | 0.027 | 55 |  |  |

**Table S7.** Landmark analysis of outcomes for TAVI compared with SAVR in participants with lower risk.

|  | **Studies** | **TAVI** | **SAVR** | **Effect Estimate** | **P value** | **I2, %** | **Subgroup difference** | |
| --- | --- | --- | --- | --- | --- | --- | --- | --- |
|  |  |  |  |  |  |  | **P value** | **x2** |
| **All-cause death or disabling stroke** |  |  |  |  |  |  |  |  |
| Within 1 year | 4 | 69/1833 | 98/1778 | 0.67 (0.49, 0.93) | 0.015 | 0 | 0.011 | 9 |
| 1 year to 2 years | 3 | 31/1375 | 18/1323 | 1.66 (0.92, 2.99) | 0.093 | 0 |  |  |
| Beyond 2 years | 3 | 116/1375 | 99/1323 | 1.17 (0.79, 1.72) | 0.433 | 42.9 |  |  |
| **All-cause death** |  |  |  |  |  |  |  |  |
| Within 1 year | 4 | 48/1833 | 69/1778 | 0.67 (0.46, 0.98) | 0.038 | 0 | 0.158 | 3.69 |
| 1 year to 2 years | 3 | 22/1375 | 20/1323 | 1.05 (0.56, 1.96) | 0.878 | 0 |  |  |
| Beyond 2 years | 3 | 103/1375 | 90/1323 | 1.12 (0.75, 1.69) | 0.574 | 43.3 |  |  |
| **Cardiovascular death** |  |  |  |  |  |  |  |  |
| Within 1 year | 4 | 35/1833 | 52/1778 | 0.65 (0.42, 1.01) | 0.054 | 0 | 0.165 | 3.61 |
| 1 year to 2 years | 3 | 11/1375 | 11/1323 | 0.96 (0.41, 2.25) | 0.92 | 0 |  |  |
| Beyond 2 years | 3 | 60/1375 | 50/1323 | 1.15 (0.77, 1.70) | 0.492 | 0 |  |  |
| **Stroke** |  |  |  |  |  |  |  |  |
| Within 1 year | 4 | 65/1833 | 61/1778 | 0.93 (0.48, 1.83) | 0..842 | 63.7 | 0.617 | 0.97 |
| 1 year to 2 years | 3 | 19/1375 | 15/1323 | 1.21 (0.6, 2.42) | 0.598 | 0 |  |  |
| Beyond 2 years | 2 | 23/641 | 14/589 | 1.51 (0.76, 2.98) | 0.237 | 0 |  |  |
| **Permanent pacemaker implantation** |  |  |  |  |  |  |  |  |
| Within 1 year | 4 | 293/1833 | 105/1778 | 3.34 (1.61, 6.9) | 0.001 | 86.7 | 0.094 | 4.74 |
| 1 year to 2 years | 3 | 26/1375 | 21/1323 | 1.19 (0.67, 2.14) | 0.549 | 0 |  |  |
| Beyond 2 years | 3 | 38/1375 | 22/1323 | 1.55 (0.58, 4.08) | 0.38 | 60.4 |  |  |
| **Rehospitalization** |  |  |  |  |  |  |  |  |
| Within 1 year | 2 | 59/1230 | 93/1188 | 0.58 (0.41, 0.82) | 0.002 | 0 | 0.005 | 10.6 |
| 1 year to 2 years | 2 | 22/1230 | 14/1188 | 1.49 (0.7, 3.2) | 0.3 | 16..2 |  |  |
| Beyond 2 years | 2 | 55/1230 | 42/1188 | 1.27 (0.84, 1.92) | 0.251 | 0 |  |  |

**Table S8.** Outcomes at 5-year follow-up for TAVI compared with SAVR stratified by types of TAVI valves.

|  |  |  |  |  |  |  | **Subgroup difference** | |
| --- | --- | --- | --- | --- | --- | --- | --- | --- |
| **Outcome or Subgroup** | **Studies** | **TAVI** | **SAVR** | **OR (95% CI)** | **P value** | **I2, %** | **P value** | **x2** |
| **All-cause death or disabling stroke** |  |  |  |  |  |  |  |  |
| Balloon-expandable | 3 | 747/1855 | 629/1826 | 1.38 (1.2, 1.6) | <0.0001 | 0 | 0.005 | 7.8 |
| Self-expanding | 4 | 602/2133 | 549/2022 | 1.03 (0.89, 1.19) | 0.66 | 0 |  |  |
| **All-cause death** |  |  |  |  |  |  |  |  |
| Balloon-expandable | 3 | 713/1855 | 602/1826 | 1.37 (1.18, 1.58) | <0.0001 | 0 | 0.014 | 6.01 |
| Self-expanding | 4 | 555/2133 | 499/2022 | 0.83 (0.58, 1.17) | 0.501 | 0 |  |  |
| **Cardiovascular death** |  |  |  |  |  |  |  |  |
| Balloon-expandable | 3 | 418/1855 | 367/1826 | 1.2 (1.02, 1.42) | 0.028 | 0 | 0.186 | 1.75 |
| Self-expanding | 4 | 337/2133 | 308/2022 | 1.02 (0.86, 1.22) | 0.805 | 0 |  |  |
| **Myocardial infarction** |  |  |  |  |  |  |  |  |
| Balloon-expandable | 3 | 99/1855 | 91/1826 | 0.75 (0.32, 1.73) | 0.498 | 76.6 | 0.157 | 2.01 |
| Self-expanding | 4 | 99/2133 | 66/2022 | 1.43 (1.04, 1.97) | 0.028 | 0 |  |  |
| **Stroke** |  |  |  |  |  |  |  |  |
| Balloon-expandable | 3 | 184/1855 | 160/1826 | 1.16 (0.93, 1.45) | 0.192 | 0 | 0.056 | 3.65 |
| Self-expanding | 3 | 155/1399 | 165/1288 | 0.85 (0.67, 1.07) | 0.162 | 0 |  |  |
| **Transient ischemic attack** |  |  |  |  |  |  |  |  |
| Balloon-expandable | 2 | 59/1359 | 40/1372 | 1.51 (1.0, 2.27) | 0.048 | 0 | 0.386 | 0.75 |
| Self-expanding | 3 | 69/1399 | 54/1288 | 1.18 (0.82, 1.71) | 0.365 | 0 |  |  |
| **Permanent pacemaker implantation** |  |  |  |  |  |  |  |  |
| Balloon-expandable | 3 | 229/1855 | 179/1826 | 1.3 (1.05, 1.6) | 0.014 | 0 | <0.0001 | 22.97 |
| Self-expanding | 4 | 623/2133 | 216/2022 | 3.56 (2.49, 5.08) | <0.0001 | 72.9 |  |  |
| **New-onset atrial fibrillation** |  |  |  |  |  |  |  |  |
| Balloon-expandable | 2 | 196/1507 | 446/1475 | 0.32 (0.19, 0.53) | <0.0001 | 84.3 | 0.383 | 0.76 |
| Self-expanding | 2 | 134/879 | 358/869 | 0.25 (0.2, 0.31) | <0.0001 | 0 |  |  |
| **Rehospitalization** |  |  |  |  |  |  |  |  |
| Balloon-expandable | 3 | 454/1855 | 364/1826 | 1.22 (0.83, 1.81) | 0.313 | 80.7 | 0.993 | 0 |
| Self-expanding | 3 | 371/1988 | 294/1887 | 1.22 (0.97, 1.54) | 0.094 | 43.9 |  |  |
| **Reintervention** |  |  |  |  |  |  |  |  |
| Balloon-expandable | 2 | 33/1507 | 18/1475 | 1.78 (0.47, 6.8) | 0.399 | 79.3 | 0.922 | 0.01 |
| Self-expanding | 4 | 49/2133 | 24/2022 | 1.92 (0.97, 3.79) | 0.06 | 31.1 |  |  |
| **Moderate to severe PVL** |  |  |  |  |  |  |  |  |
| Balloon-expandable | 2 | 23/1497 | 1/1458 | 14.2 (2.7, 75.0) | 0.002 | 0 | 0.475 | 0.51 |
| Self-expanding | 4 | 26/2110 | 2/2000 | 6.84 (2.2, 21.1) | 0.001 | 0 |  |  |

**Table S9.** Landmark analysis of outcomes for TAVI with balloon-expandable valves compared with SAVR.

|  | **Studies** | **TAVI** | **SAVR** | **Effect Estimate** | **P value** | **I2, %** | **Subgroup difference** | |
| --- | --- | --- | --- | --- | --- | --- | --- | --- |
|  |  |  |  |  |  |  | **P value** | **x2** |
| **All-cause death or disabling stroke** |  |  |  |  |  |  |  |  |
| Within 1 year | 3 | 242/1855 | 266/1826 | 0.87 (0.64, 1.18) | 0.376 | 44.7 | 0.004 | 10.8 |
| 1 year to 2 years | 3 | 92/1855 | 72/1826 | 1.29 (0.94, 1.78) | 0.116 | 0 |  |  |
| Beyond 2 years | 3 | 413/1855 | 291/1826 | 1.57 (1.32, 1.86) | <0.0001 | 0 |  |  |
| **All-cause death** |  |  |  |  |  |  |  |  |
| Within 1 year | 4 | 212/1855 | 224/1826 | 0.93 (0.14, 1.2) | 0.571 | 21.5 | 0.004 | 10.8 |
| 1 year to 2 years | 3 | 82/1855 | 74/1826 | 1.11 (0.8, 1.54) | 0.517 | 0 |  |  |
| Beyond 2 years | 3 | 419/1855 | 304/1826 | 1.52 (1.29, 1.8) | <0.0001 | 0 |  |  |
| **Cardiovascular death** |  |  |  |  |  |  |  |  |
| Within 1 year | 3 | 121/1855 | 126/1826 | 0.95 (0.65, 1.38) | 0.775 | 37.6 | 0.21 | 3.12 |
| 1 year to 2 years | 3 | 51/1855 | 49/1826 | 1.04 (0.7, 1.56) | 0.829 | 0 |  |  |
| Beyond 2 years | 3 | 246/1855 | 192/1826 | 1.32 (1.09, 1.65) | 0.005 | 0 |  |  |
| **Stroke** |  |  |  |  |  |  |  |  |
| Within 1 year | 3 | 104/1855 | 103/1826 | 0.98 (0.47, 2.04) | 0.951 | 72.2 | 0.4 | 1.83 |
| 1 year to 2 years | 3 | 23/1855 | 12/1826 | 1.88 (0.92, 3.83) | 0.084 | 0 |  |  |
| Beyond 2 years | 3 | 57/1855 | 45/1826 | 1.08 (0.51, 2.27) | 0.849 | 64.6 |  |  |
| **Permanent pacemaker implantation** |  |  |  |  |  |  |  |  |
| Within 1 year | 3 | 153/1855 | 125/1826 | 1.23 (0.96, 1.57) | 0.1 | 0 | 0.869 | 0.28 |
| 1 year to 2 years | 3 | 28/1855 | 20/1826 | 1.38 (0.77, 2.46) | 0.276 | 0 |  |  |
| Beyond 2 years | 3 | 48/1855 | 34/1826 | 1.38 (0.89, 2.16) | 0.153 | 0 |  |  |
| **Rehospitalization** |  |  |  |  |  |  |  |  |
| Within 1 year | 3 | 236/1855 | 229/1826 | 1.0 (0.69, 1.44) | 0.987 | 65.9 | 0.099 | 4.62 |
| 1 year to 2 years | 3 | 63/1855 | 42/1826 | 1.44 (0.88, 2.35) | 0.148 | 25.8 |  |  |
| Beyond 2 years | 3 | 155/1855 | 93/1826 | 1.68 (1.24, 2.28) | 0.001 | 17.7 |  |  |

**Table S10.** Landmark analysis of outcomes for TAVI with self-expanding valves compared with SAVR.

|  | **Studies** | **TAVI** | **SAVR** | **Effect Estimate** | **P value** | **I2, %** | **Subgroup difference** | |
| --- | --- | --- | --- | --- | --- | --- | --- | --- |
|  |  |  |  |  |  |  | **P value** | **x2** |
| **All-cause death or disabling stroke** |  |  |  |  |  |  |  |  |
| Within 1 year | 4 | 163/2133 | 195/2022 | 0.75 (0.6, 0.94) | 0.012 | 0 | 0.015 | 8.47 |
| 1 year to 2 years | 4 | 93/2133 | 79/2022 | 1.1 (0.81, 1.5) | 0.551 | 0 |  |  |
| Beyond 2 years | 4 | 346/2133 | 275/2022 | 1.21 (0.93, 1.56) | 0.15 | 46.4 |  |  |
| **All-cause death** |  |  |  |  |  |  |  |  |
| Within 1 year | 4 | 133/2133 | 147/2022 | 0.82 (0.64, 1.06) | 0.125 | 0 | 0.125 | 4.17 |
| 1 year to 2 years | 4 | 87/2133 | 77/2022 | 1.05 (0.77, 1.45) | 0.751 | 0 |  |  |
| Beyond 2 years | 4 | 335/2133 | 275/2022 | 1.16 (0.93, 1.43) | 0.188 | 25.2 |  |  |
| **Cardiovascular death** |  |  |  |  |  |  |  |  |
| Within 1 year | 4 | 99/2133 | 116/2022 | 0.78 (0.59, 1.03) | 0.082 | 0 | 0.086 | 4.9 |
| 1 year to 2 years | 4 | 49/2133 | 38/2022 | 1.17 (0.63, 2.19) | 0.617 | 39.1 |  |  |
| Beyond 2 years | 4 | 189/2133 | 154/2022 | 1.16 (0.92, 1.45) | 0.201 | 0 |  |  |
| **Stroke** |  |  |  |  |  |  |  |  |
| Within 1 year | 4 | 112/2133 | 131/2022 | 0.79 90.61, 1.03) | 0.078 | 0 | 0.066 | 5.44 |
| 1 year to 2 years | 4 | 26/2133 | 34/2022 | 0.73 (0.43, 1.22) | 0.229 | 0 |  |  |
| Beyond 2 years | 3 | 60/1399 | 41/1288 | 1.35 (0.9, 2.03) | 0.146 | 0 |  |  |
| **Permanent pacemaker implantation** |  |  |  |  |  |  |  |  |
| Within 1 year | 3 | 277/1269 | 86/1226 | 4.63 (2.09, 10.3) | <0.0001 | 85.2 | 0.033 | 6.8 |
| 1 year to 2 years | 3 | 29/1269 | 19/1226 | 1.45 (0.8, 2.64) | 0.219 | 0 |  |  |
| Beyond 2 years | 4 | 58/2133 | 42/2022 | 1.3 (0.66, 2.53) | 0.448 | 49.9 |  |  |
| **Rehospitalization** |  |  |  |  |  |  |  |  |
| Within 1 year | 3 | 157/1988 | 149/1887 | 0.93 (0.56, 1.54) | 0.772 | 76.6 | 0.011 | 9 |
| 1 year to 2 years | 3 | 72/1988 | 30/1887 | 2.29 (1.49, 3.5) | <0.0001 | 0 |  |  |
| Beyond 2 years | 3 | 142/1988 | 115/1887 | 1.17 (0.91, 1.51) | 0.232 | 0 |  |  |

**Table S11.** Outcomes at 5-year follow-up for TAVI compared with SAVR stratified by surgical risk.

|  |  |  |  |  |  |  | **Subgroup difference** | |
| --- | --- | --- | --- | --- | --- | --- | --- | --- |
| **Outcome or Subgroup** | **Studies** | **TAVI** | **SAVR** | **OR (95% CI)** | **P value** | **I2, %** | **P value** | **x2** |
| **All-cause death** |  |  |  |  |  |  |  |  |
| Higher risk | 4 | 1116/2614 | 952/2525 | 1.26 (1.12, 1.42) | 0.0002 | 6 | 0.11 | 59.9 |
| Lower risk | 3 | 152/1375 | 149/1323 | 0.98 (0.74, 1.30) | 0.91 | 22 |  |  |
| **Cardiovascular death** |  |  |  |  |  |  |  |  |
| Higher risk | 4 | 662/2614 | 577/2525 | 1.16 (1.02, 1.32) | 0.026 | 0 | 0.125 | 2.36 |
| Lower risk | 3 | 93/1375 | 98/1323 | 0.9 (0.66, 1.21) | 0.479 | 0 |  |  |
| **Myocardial infarction** |  |  |  |  |  |  |  |  |
| Higher risk | 4 | 144/2613 | 112/2525 | 1.21 (0.85, 1.72) | 0.283 | 30.8 | 0.738 | 0.11 |
| Lower risk | 3 | 54/1375 | 45/1323 | 1.04 (0.44, 2.42) | 0.936 | 73.9 |  |  |
| **Stroke** |  |  |  |  |  |  |  |  |
| Higher risk | 4 | 299/2613 | 288/2525 | 0.99 (0.78, 1.25) | 0.904 | 41.3 | 0.976 | 0 |
| Lower risk | 2 | 40/641 | 37/589 | 0.99 (0.63, 1.58) | 0.978 | 0 |  |  |
| **Transient ischemic attack** |  |  |  |  |  |  |  |  |
| Higher risk | 4 | 119/2613 | 89/2525 | 1.3 (0.98, 1.72) | 0.07 | 0 | 0.632 | 0.23 |
| Lower risk | 1 | 9/145 | 5/135 | 1.72 (0.56, 5.27) | 0.342 | na |  |  |
| **Permanent pacemaker implantation** |  |  |  |  |  |  |  |  |
| Higher risk | 4 | 560/2613 | 280/2525 | 1.95 (1.08, 3.51) | 0.026 | 91.8 | 0.339 | 0.92 |
| Lower risk | 3 | 292/1375 | 115/1323 | 3.21 (1.39, 7.42) | 0.006 | 90.4 |  |  |
| **New-onset atrial fibrillation** |  |  |  |  |  |  |  |  |
| Higher risk | 1 | 141/1011 | 291/1021 | 0.41 (0.33, 0.51) | <0.0001 | na | 0.001 | 11.3 |
| Lower risk | 3 | 189/1375 | 513/1323 | 0.25 (0.2, 0.3) | <0.0001 | 0 |  |  |
| **Rehospitalization** |  |  |  |  |  |  |  |  |
| Higher risk | 4 | 689/2613 | 509/2525 | 1.43 (1.25, 1.63) | <0.0001 | 0 | <0.0001 | 12.6 |
| Lower risk | 2 | 136/1230 | 149/1188 | 0.86 (0.67, 1.1) | 0.227 | 0 |  |  |
| **Reintervention** |  |  |  |  |  |  |  |  |
| Higher risk | 3 | 58/2265 | 19/2174 | 2.9 (1.72, 4.9) | <0.0001 | 0 | 0.007 | 7.4 |
| Lower risk | 3 | 24/1375 | 23/1323 | 0.98 (0.55, 1.75) | 0.939 | 0 |  |  |
| **Moderate to severe PVL** |  |  |  |  |  |  |  |  |
| Higher risk | 3 | 37/2244 | 3/2158 | 9.0 (2.9, 27.3) | <0.0001 | 0 | 0.898 | 0.02 |
| Lower risk | 3 | 12/1363 | 0/1300 | 7.83 (1.42, 43.2) | 0.018 | 0 |  |  |

**Figure S1.** PRISMA flow diagram

## Included

## Identification

## Screening

## Eligibility

Full-text articles excluded, with reasons (n= 31):

Secondary report without eligible data (n= 7)

Not comparing TAVI versus SAVR (n= 10)

Not randomized controlled trial (n= 14)

Trials included in quantitative synthesis (meta-analysis) (n = 8)

Secondary reports with eligible data (n = 14)

Full-text articles assessed for eligibility
(n = 53)

Additional records identified through other sources
(n = 21 )

Records identified through database searching
(n = 3027)

Records excluded
(n = 1871)

Records screened
(n = 1924)

Records after duplicates removed
(n = 1924)

**Figure S2.** Risk estimates of all-cause death for TAVI vs SAVR stratified by surgical risks at different lengths of follow-up.

**
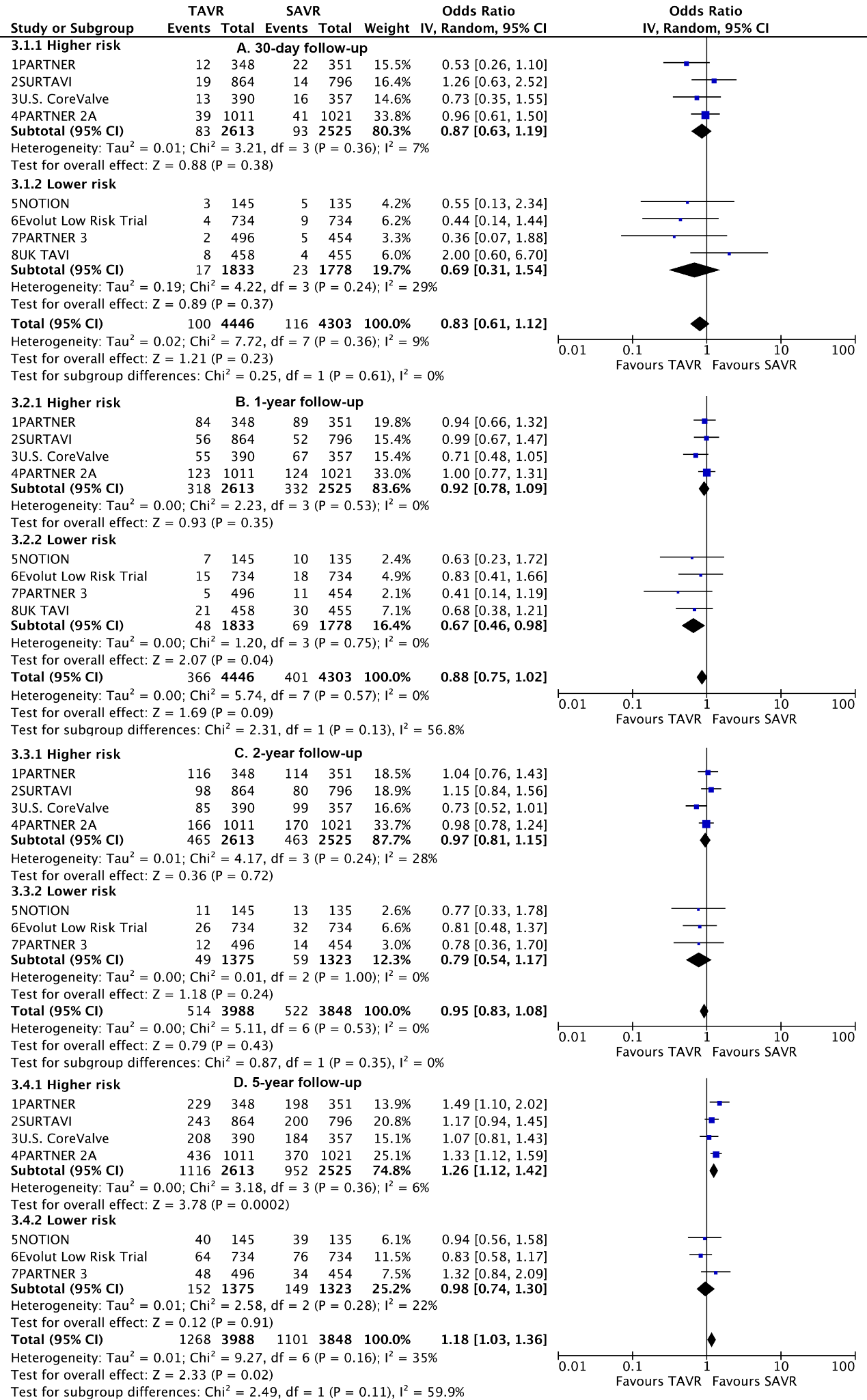
**

**Figure S3.** Risk estimates of all-cause death for TAVI vs SAVR stratified by surgical risks according to different timing intervals.

**
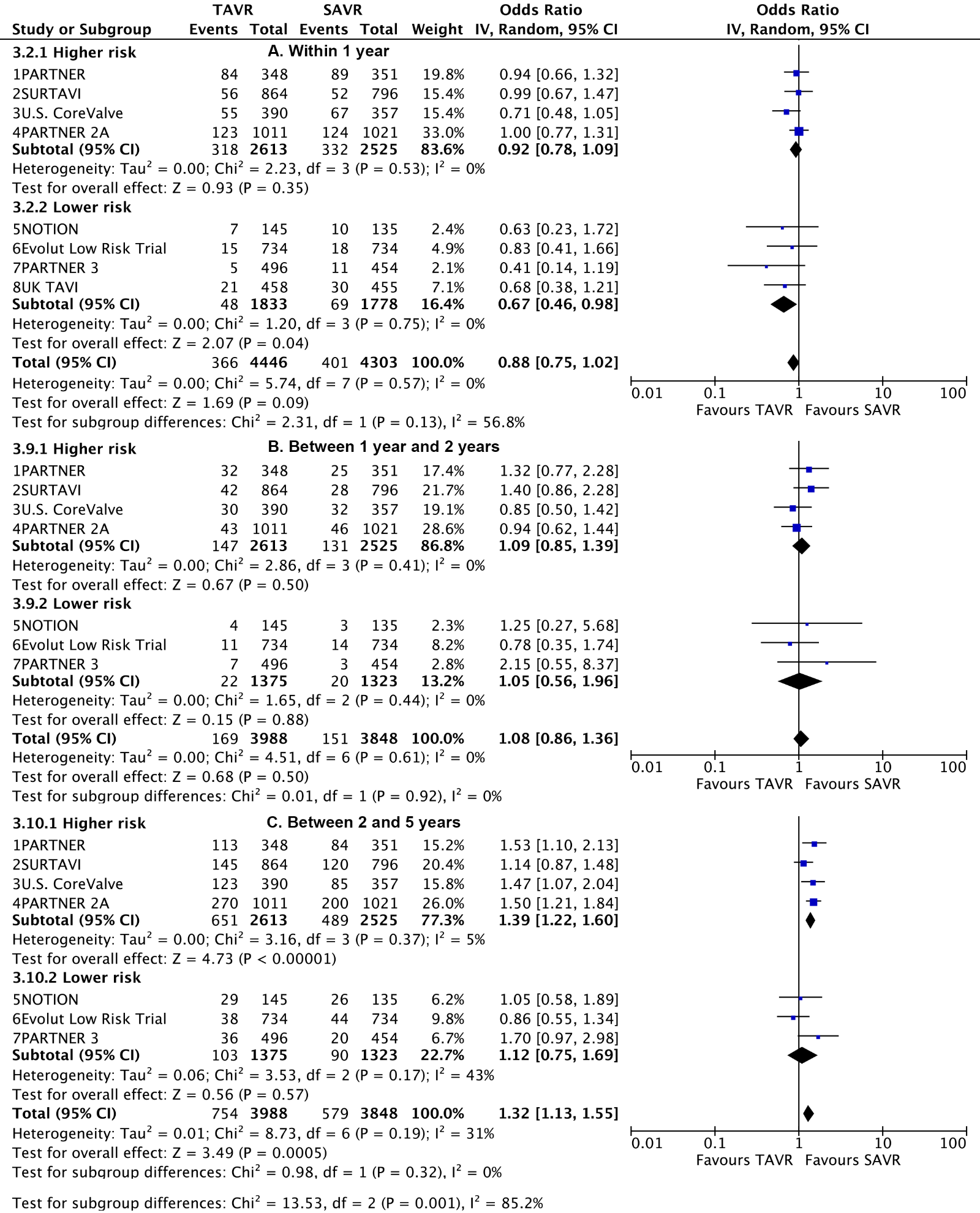
**

**Figure S4.** Sensitivity analysis for risk of all-cause death or disabling stroke stratified by lengths of follow-up, with the HKSJ model.

**
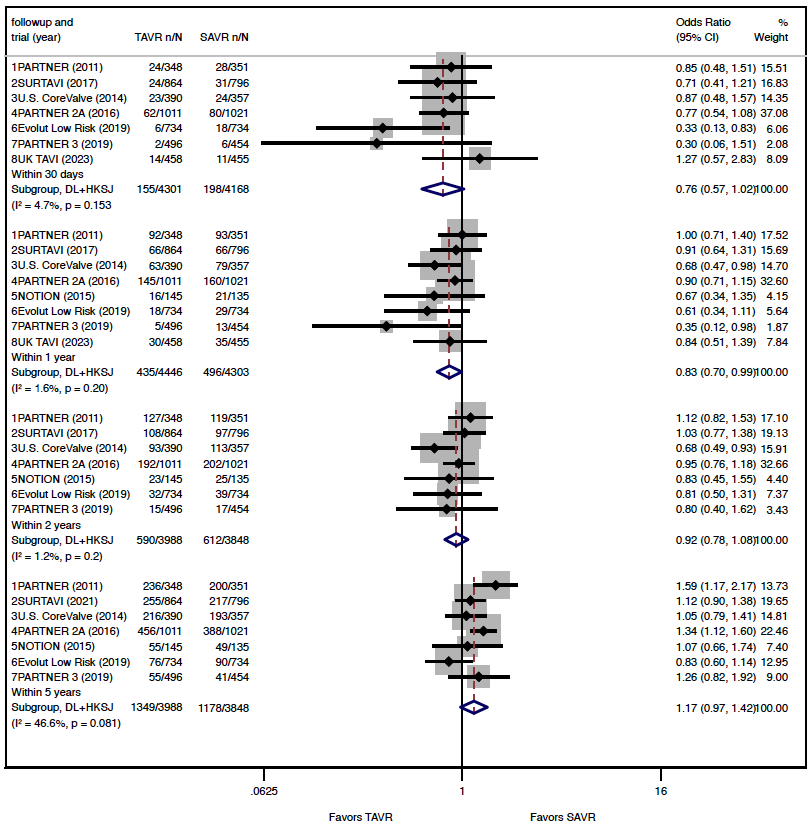
**

**Figure S5.** Sensitivity analysis for risk of all-cause death or disabling stroke according to different timing intervals, with the HKSJ model.

**
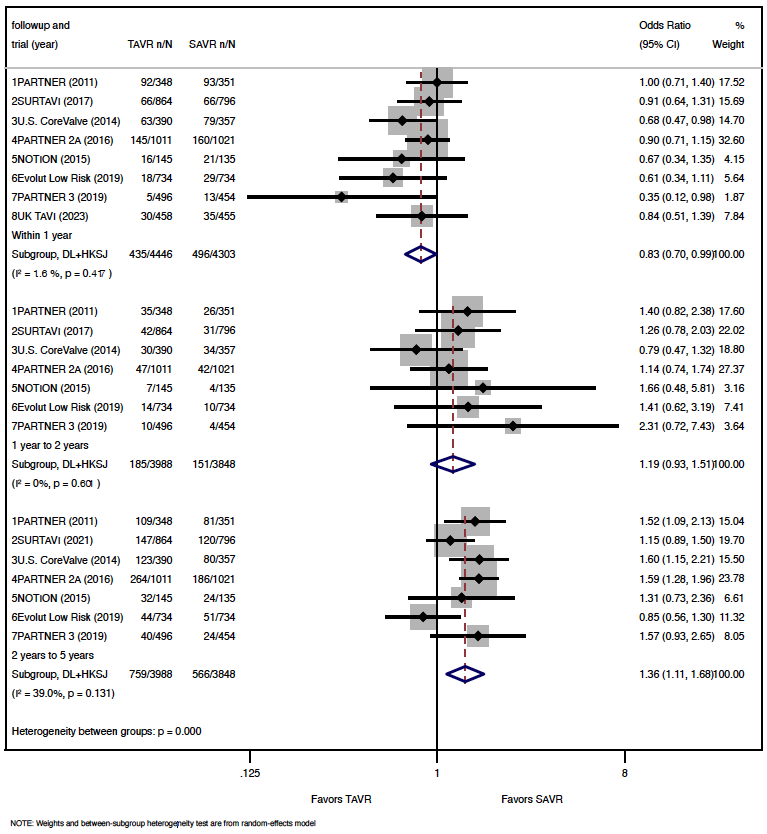
**

**Figure S6**. Sensitivity analysis for the risk of all-cause death or disabling stroke stratified by lengths of follow-up after removing each trial one-by one

**
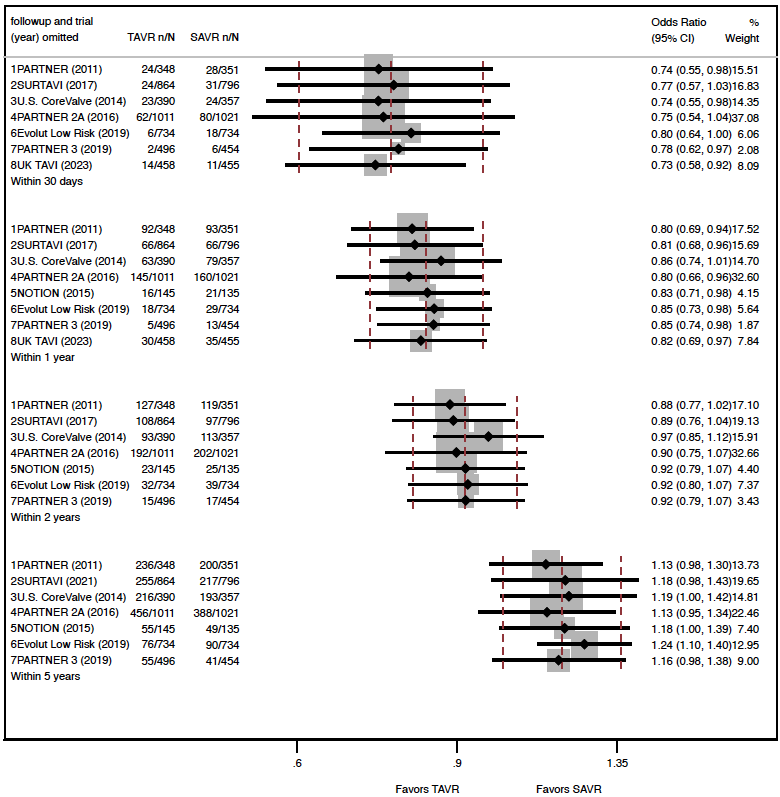
**

**Figure S7.** Sensitivity analysis for the risk of all-cause death or disabling stroke according to different timing after removing each trial one-by one

**
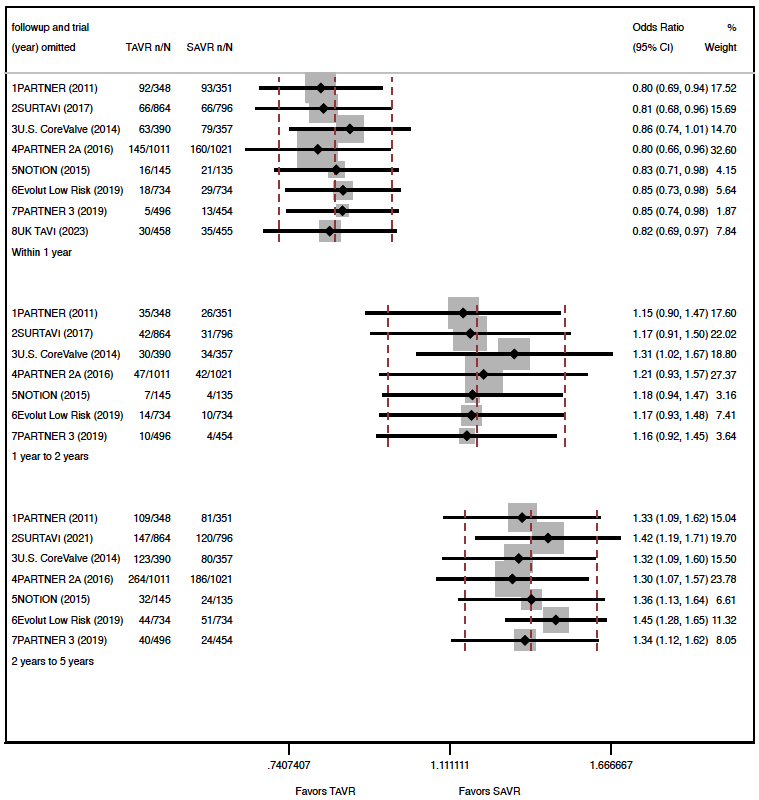
**
